# Uncovering the cellular and molecular changes in tendon stem/progenitor cells attributed to tendon aging and degeneration

**DOI:** 10.1111/acel.12124

**Published:** 2013-07-22

**Authors:** Julia Kohler, Cvetan Popov, Barbara Klotz, Paolo Alberton, Wolf Christian Prall, Florian Haasters, Sigrid Müller-Deubert, Regina Ebert, Ludger Klein-Hitpass, Franz Jakob, Matthias Schieker, Denitsa Docheva

**Affiliations:** 1 Department of Surgery, Experimental Surgery and Regenerative Medicine, Ludwig Maximilians University MunichNussbaumstr. 20, 80336, Munich, Germany; 2 Orthopedic Centre for Musculoskeletal Research, Julius Maximilians UniversityBrettreichstr. 11, 97074, Wuerzburg, Germany; 3 Institute of Cell Biology (Cancer Research), Medical Faculty, University of Duisburg-EssenVirchowstr. 173, 45122, Essen, Germany

**Keywords:** actin dynamics, cell–matrix interactions, microarray, ROCK kinase, senescence, tendon stem/progenitor cells

## Abstract

Although the link between altered stem cell properties and tissue aging has been recognized, the molecular and cellular processes of tendon aging have not been elucidated. As tendons contain stem/progenitor cells (TSPC), we investigated whether the molecular and cellular attributes of TSPC alter during tendon aging and degeneration. Comparing TSPC derived from young/healthy (Y-TSPC) and aged/degenerated human Achilles tendon biopsies (A-TSPC), we observed that A-TSPC exhibit a profound self-renewal and clonogenic deficits, while their multipotency was still retained. Senescence analysis showed a premature entry into senescence of the A-TSPC, a finding accompanied by an upregulation of p16^INK4A^. To identify age-related molecular factors, we performed microarray and gene ontology analyses. These analyses revealed an intriguing transcriptomal shift in A-TSPC, where the most differentially expressed probesets encode for genes regulating cell adhesion, migration, and actin cytoskeleton. Time-lapse analysis showed that A-TSPC exhibit decelerated motion and delayed wound closure concomitant to a higher actin stress fiber content and a slower turnover of actin filaments. Lastly, based on the expression analyses of microarray candidates, we suggest that dysregulated cell–matrix interactions and the ROCK kinase pathway might be key players in TSPC aging. Taken together, we propose that during tendon aging and degeneration, the TSPC pool is becoming exhausted in terms of size and functional fitness. Thus, our study provides the first fundamental basis for further exploration into the molecular mechanisms behind tendon aging and degeneration as well as for the selection of novel tendon-specific therapeutical targets.

## Introduction

With advancing age, tendons become more prone to tissue degeneration and subsequent injury (Tuite *et al*., 1997; Smith *et al*., 2002; Rees *et al*., 2006). Tendon repair requires lengthy periods of rehabilitation particularly in elderly patients. Furthermore, existing medical and surgical treatments often fail to restore the initial tendon strength. In the past decade, tremendous progress has been made in understanding the developmental and cell biology of tendons. Nevertheless, to date, the underlying molecular and cellular mechanisms of tendon aging and degeneration remain largely unknown.

Aging is defined as an overall decline in the functional capacity of various organs to maintain tissue homeostasis, accompanied by a diminished regenerative response (Liu & Rando, 2011). Several studies of human aging and age-associated diseases such as sarcopenia, dementia, cancer, or impaired wound healing indicate that the reduced regenerative potential of adult tissues is linked to a functional decline of their stem cell pool (Rando, 2006; Sahin & Depinho, 2010). Thus, it is now assumed that aging is driven in part by an age-associated shortage in number, stress resistance, and repair capacity of tissue-specific adult stem cells (Sharpless & DePinho, 2007).

Mesenchymal stem cells (MSC) are adult stem cells found in bone marrow and other tissues. MSC are capable of mobilizing, proliferating, and committing to terminally differentiated cell types. Thus, MSC are a potent cell source for tissue regeneration (Docheva *et al*., 2007; Bianco *et al*., 2010). Recent investigations focusing on MSC from bone marrow showed apparent age-related changes including reduced proliferation and clonogenicity as well as altered differentiation potential (Baxter *et al*., 2004; Stolzing & Scutt, 2006; Stolzing *et al*., 2008; Kasper *et al*., 2009; Yu *et al*., 2011). Furthermore, several other studies have shown increased levels of senescence-associated genes encoded by the CDKN2a locus (p14^ARF^, p16^INK4A^) in MSC from aged bone marrow aspirates (Shibata *et al*., 2007; Stolzing *et al*., 2008). Kasper *et al*. (2009) reported a connection between tissue aging, MSC stress resistance, and regenerative potential; specifically, fewer MSC were observed with age, and they exhibited augmented senescence, reduced antioxidant defense, changes in cytoskeletal organization, and lower migratory capacity. Taken together, these studies showed that like other somatic stem cells, MSC experience common mechanisms of cell aging, which impair their functions.

In 2007, Bi *et al*. identified a population of residing tendon stem/progenitor cells (TSPC) within human hamstring tendons. They showed that the TSPC exhibit classical MSC characteristics, having typical surface antigens, self-renewal, clonogenicity, and three-lineage differentiation capacity. Yet, in contrast to MSC, TSPC express tendon-related genes such as scleraxis and tenomodulin and are able to form tendon and enthesis-like tissues when implanted *in vivo*. The existence of TSPC was further confirmed in subsequent studies with tendons from different species (Tempfer *et al*., 2009; Zhou *et al*., 2010; Haasters *et al*., 2011; Mienaltowski *et al*., 2013).

To further decipher tissue aging and age-associated conditions, in this study we focused on human TSPC, comparing cells derived from Achilles tendon biopsies of young and healthy individuals (Y-TSPC) with those of aged patients with functionally degenerated tissues (A-TSPC). Using microarray analysis and different functional readouts, we investigated whether TSPC alter their molecular and cellular characteristics during aging and degeneration. For the first time to our knowledge, this study identifies the distinct molecular and cellular changes in TSPC that contribute to human tendon aging and degeneration.

## Results

**Fig 1:**
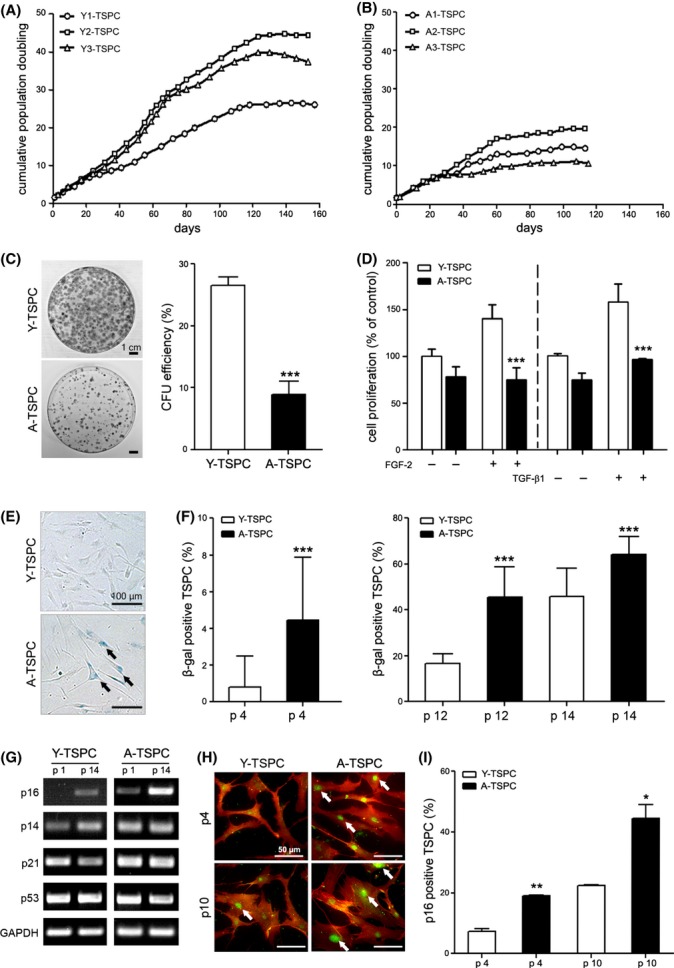
Short- and long-term proliferation analyses and investigation of cellular senescence. (A and B) Growth curves of three different donors per TSPC group were assembled by calculating cumulative population doubling time. (C) Colony-forming unit (CFU) assay. Crystal violet-stained colonies at day 14. CFU efficiency shown as mean ± SD of three Y-TSPC and three A-TSPC from two independent experiments. (D) Proliferation analysis prior and after stimulation with FGF-2 (1 ng mL^−1^, 7 days) and TGF-β_1_ (50 ng mL^−1^, 3 day). Two independent WST-1 measurements with three different donors per group were performed. (E) Senescence-associated β-gal staining at passage 12 (arrows indicate senescence cells). (F) Quantification of senescent cell number at passage 4, 12, and 14. Three Y- and three A-TSPC donors were analyzed as approx. 600 cells per donor were evaluated. (G) Semi-quantitative PCR analysis of cell cycle gene regulators (p16^INKA4^, p14^ARF^, p21^WAF1^, and p53) in TSPC in passages 1, 10, and 14. PCR was carried out twice independently with three different donors per group. Staining for p16^INKA4^ (in green) and CD44 (in red). (I) Quantification of p16^INKA4^-positive cells. Two different donors (200 cells/donor) per group were analyzed at two different passages.

TSPC were successfully isolated from human Achilles tendon biopsies (Table S1) and characterized *in vitro* to validate their stem/progenitor character. We used FACS and immunocytochemistry to examine the expression of surface antigens and stem cell markers in TSPC based on the studies by Bi *et al*. (2007), Tempfer *et al*. (2009), and Mienaltowski *et al*. (2013). More than 98% of Y-TSPC and A-TSPC were positive for MSC-related surface antigens CD73, CD90, and CD105 (Fig. S1A). They were negative for hematopoietic and endothelial cell antigens CD19, CD34, CD45, and HLA-DRA, thus excluding possible contamination with such cells (Table S2 and S3). TSPC were also positive for STRO-1 (MSC marker), CD146 (pericyte marker), Musashi-1 (muscle, neural, and pericyte markers) (Fig. S1B), and CD44 (MSC marker, Fig.1H). Next, we performed quantitative and semi-quantitative PCR analyses of tendon-related genes, which confirmed the expression of the transcription factors scleraxis, Eya1, and Six1, the tendon marker gene tenomodulin and several extracellular matrix proteins abundant in tendon (collagen types I and III, COMP, decorin, and tenascin C) in both TSPC types (Fig. S1C-E).

### TSPC from aged patients exhibit proliferation deficits accompanied by premature entry into cellular senescence

To assess potential age-related changes in the TSPC self-renewal ability, we carried out long-term proliferation and clonogenicity analyses. The Y-TSPC did not enter a growth plateau before 120 days in culture, and their PD ranged between 26 and 45 times (Fig.1A). In contrast, the A-TSPC went into an early plateau phase after approx. 60 days in culture, and their PD did not exceed 18 times (Fig.1B). Next, we conducted colony-forming unit (CFU) assays at three different platting densities and detected a significantly lower colony number and CFU efficiency in the A-TSPC compared with the Y-TSPC (Fig.1C and Fig. S2). To further verify the observed self-renewal deficit, we analyzed the proliferative activity of the TSPC, with or without FGF-2 and TGF-β1 stimulation, using a WST-1 assay. We selected FGF-2 because it is a strong mitogen for a wide variety of cells and TGF-β1 because it is essential for the recruitment and maintenance of early tendon progenitors (Pryce *et al*., 2009) as well as it is a potent stimulant of MSC proliferation (Ng *et al*., 2008). The WST-1 results confirmed the age-dependent aberrance in cell proliferation as they revealed a significantly declined proliferative activity and a poor response to FGF-2 and TGF-β1 stimulation in the A-TSPC (Fig.1D).

To examine whether A-TSPC experience an early onset of cellular senescence, we quantified at three different time points the number of senescent cells using β-galactosidase (β-gal) staining (Fig.1E,F). Already at passage 4, the A-TSPC populations possessed 5-fold more senescent cells when compared to the Y-TSPC. Moreover, the number of β-gal-positive cells in A-TSPC remained significantly higher also in later passages (Fig.1F). The progression of cell senescence and apoptosis has been directly linked to elevated expression of cell cycle repressor genes such as p14^ARF^, p16^INK4A^, p21^WAF1^, and p53 (Finkel *et al*., 2007; Sharpless & DePinho, 2007). Therefore, we performed a semi-quantitative PCR analysis for these genes at two different time points (Fig.1G). The results showed similar RNA levels of p14^ARF^, p21^WAF1^, and p53 in both Y-and A-TSPC. In contrast, p16^INK4A^ was expressed in the A-TSPC already at passage 1 and its expression further increased at passage 14. In the Y-TSPC, a slight p16^INK4A^ expression was detected only in the later passage. The p16^INKA4^ upregulation in A-TSPC was also confirmed on protein level by immunocytochemical staining (Fig.1H) and quantification of the number of p16^INKA4^-expressing cells (Fig.1I).

Taken together, our results clearly indicate that A-TSPC have a reduced self-renewal potential, which was apparent during short- and long-term cultivation but also when the cells were stimulated with FGF-2 or TGF-β1. We suggest that this phenomenon is due to an earlier entry of A-TSPC into cellular senescence, which we confirmed by demonstrating that the A-TSPC group exhibited a significantly higher number of senescent cells along with a substantial p16^INKA4^ upregulation.

### Aging does not abolish TSPC multipotency

Multilineage differentiation is another feature of stem/progenitor cells. Therefore, we compared TSPC capacity to undergo adipogenic, osteogenic, and chondrogenic differentiation (Fig. S3). Regarding adipogenic and osteogenic differentiation, there were no obvious differences between the groups and the individual donors. Regarding chondrogenic differentiation, pronounced donor variability was observed in each group; however, both Y- and A-TSPC produced proteoglycan-rich matrices and the cartilage marker aggrecan. In summary, we concluded that TSPC multipotency is not strongly impaired upon aging.

### Transcriptome profiling of young and aged TSPC demonstrates intriguing differences

**Fig 2:**
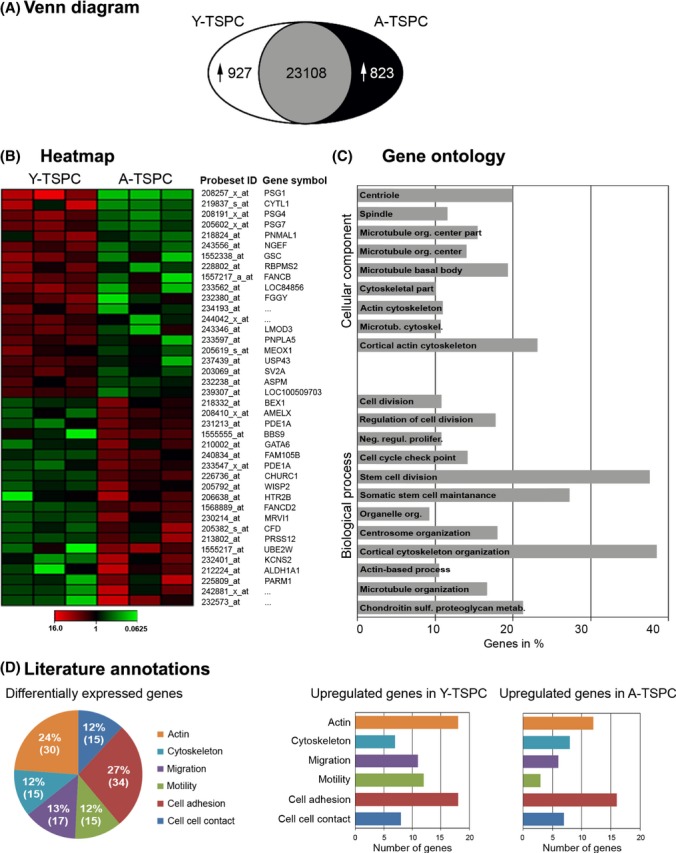
Genome-wide microarray and gene ontology analysis of differentially expressed probesets. (A) Venn diagram of global probesets changes. The number of significantly upregulated probesets in Y-TSPCand A-TSPC is shown. The intersection indicates the number of probesets that are not significantly regulated. Differentially expressed probesets were identified by pairwise comparison analysis (MAS5 algorithm) of experimental versus baseline array that displayed a signal log2 ratio of < −1 or > 1, containing a fold change (FC) lower than 0.5 and greater than 2. The detection p-value was ≤ 0.05, which was evaluated against certain cut-offs to determine the detection call (A, absent; M, marginal; P, present). The number of ‘present’ calls for a given probeset had to be greater than 66% in at least one of the donor groups. (B) Heatmap of the top 40 differentially expressed probesets. (C) Gene ontology (GO) analysis of all differentially expressed probesets identified significantly enriched ‘cellular component’ and ‘biological process’ GO clusters. (D) Cake diagram representing the literature-based categorization of the top 130 differentially expressed genes. Bar charts demonstrate for each category in (D) the exact number of upregulated genes in Y-TSPC (left) and A-TSPC (right). The data include three different donors per group.

To identify molecular factors involved in tendon aging and degeneration, we performed microchip hybridization with RNA from three different donors per group. Comparative microarray analysis is summarized in a Venn diagram in Fig.2A, and the top 40 and 100 probesets are depicted in heatmaps in Fig.2B and Fig. S4, respectively (complete microarray data in Table S4). To identify a molecular pattern behind the observed transcriptomal shift, we performed gene ontology (GO) analysis with all differentially expressed probesets. In Fig.2C, exemplary entries from the ‘cellular component’ and ‘biological process’ gene clusters are shown. Finally, to spot the most relevant genes, we applied literature-based annotation approach as follows: using probesets with 2-fold changes, 130 known genes were identified and subjected to literature screening. Our investigation demonstrated that these genes distributed majorly in the following categories: (i) ‘cell–cell contact’, (ii) ‘cell adhesion’, (iii) ‘motility’, (iv) ‘migration’, (v) ‘cytoskeleton’, and (vi) ‘actin-related transcripts’ (Fig.2D and Table S5).

For the first time to our knowledge, our microarray analysis of patient-derived TSPC reports transcriptomal alterations that are specific for tendon aging and degeneration. Moreover, it strongly suggests that A-TSPC will exhibit further phenotypic and behavioral differences to Y-TSPC. Therefore, in the following part of the study, we compared the migratory activity, actin organization, and turnover as well as the expression of several microarray gene candidates in Y- and A-TSPC.

### Aged TSPC have profoundly reduced migratory ability which is concomitant to slower actin cytoskeleton dynamics

**Fig 3:**
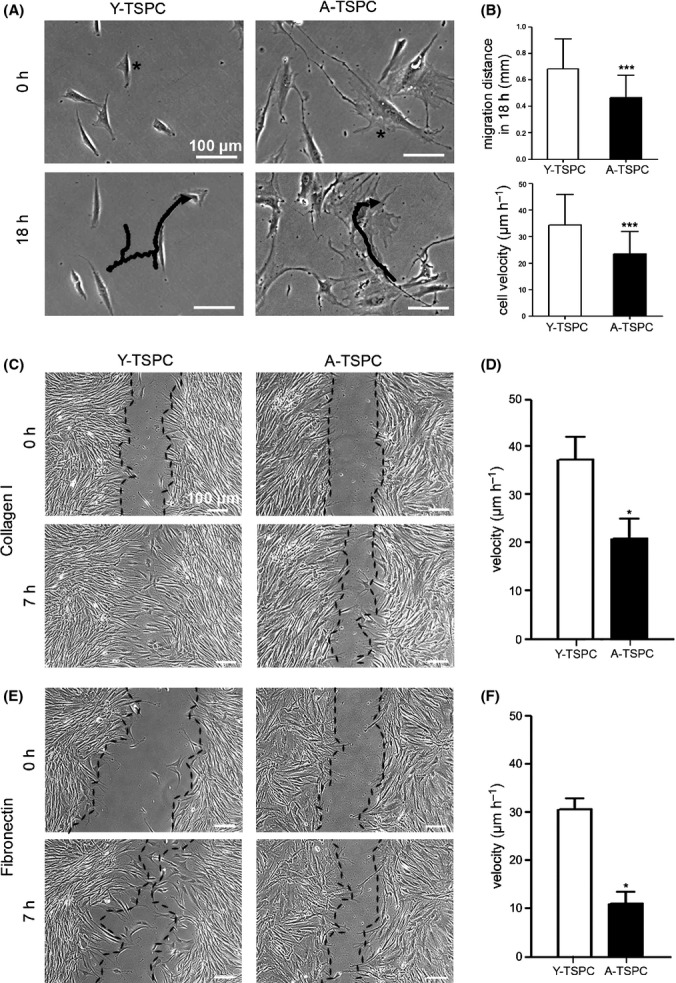
Investigation of TSPC migration potential. (A) Time-lapse experiment for 18 h. Representative images at the beginning and at the end of the experiment are shown. Tracked cells and migratory paths are indicated with stars and black lines. (B) Quantification of migration distance and cell velocity. Two independent experiments with three donors per group were performed (180 cells per group). Scratch assays on collagen I (C and D) and fibronectin (E and F). Representative images at 0 h and 7 h are shown, and the cellular fronts are outlined with black lines. Cell velocity was calculated from four different scratches per donor.

**Fig 4:**
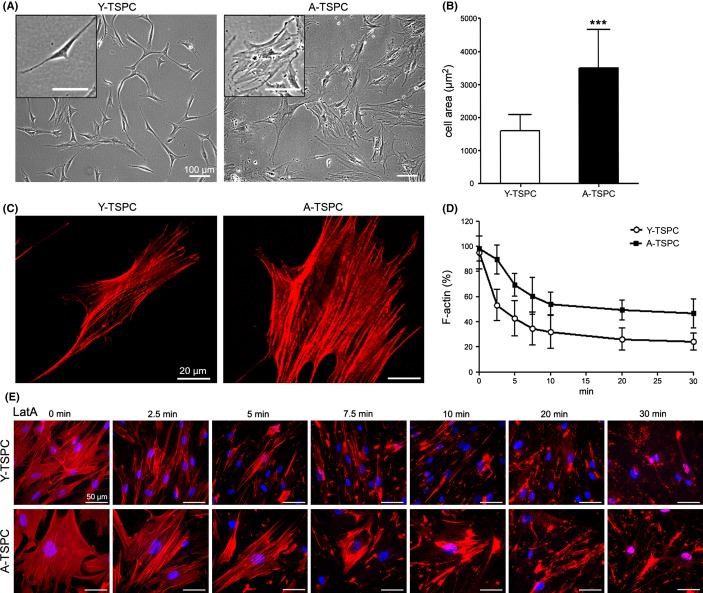
Comparison of cell size, F-actin organization, and turnover in Y- and A-TSPC. (A) Phase-contrast images at passage 1. (B) Quantification of cell area of three Y-TSPC versus three A-TSPC donors (50 cells per group) grown on plastic dishes. (C) Phalloidin-stained TSPC (actin fibers are in red). (D) Quantification of the F-actin amount in Y- and A-TSPC after Latrunculin A (LatA) treatment. The individual data points show mean ± SD from three different donors per group. (E) Representative images of phalloidin labeling at each time point of the LatA treatment. DAPI (in blue) was used for nuclear staining.

We carried out time-lapse experiments for monitoring of random cell migration and an *in vitro* scratch assay mimicking wound closure. Quantifications of migratory distance revealed that A-TSPC migration speed and distances were significantly slower compared with Y-TSPC (Fig.3A,B). To estimate the effect of matrix proteins, scratch assay experiments were performed on collagen I or fibronectin and also revealed a decelerated migration and longer wound closure time in the aged cells (Fig.3C–F). In addition, pronounced morphological differences were noticed between Y- and A-TSPC; cells from aged donors exhibited a star-like flattened cell appearance, while cells from young donors were smaller in size and spindle-shaped (Fig.4A,B). It is known that cell shape and cell migration strongly dependent on actin cytoskeleton organization and the rate of actin filament turnover (Rottner & Stradal, 2011). Therefore, we performed phalloidin stainings for F-actin and compared the actin filament dynamics by treating the TSPC with latrunculin A (LatA) in a time-dependent manner. LatA inhibits actin polymerization by sequestering monomeric G-actin and thereby disrupts the turnover of actin filaments. Our results showed that A-TSPC have more robust actin stress fibers (Fig.4C) and a higher actin content than Y-TSPC (Fig.4D,E). In conclusion, the smaller effect of LatA on the A-TSPC indicated a slower actin turnover in these cells. Taken together, our results clearly demonstrate a dramatic decrease in the migratory capacity of TSPC during aging and suggested that distorted actin dynamics might be a core reason.

### Altered extracellular matrix production, integrin expression, and ROCK activity are associated with TSPC from aged patients

**Fig 5:**
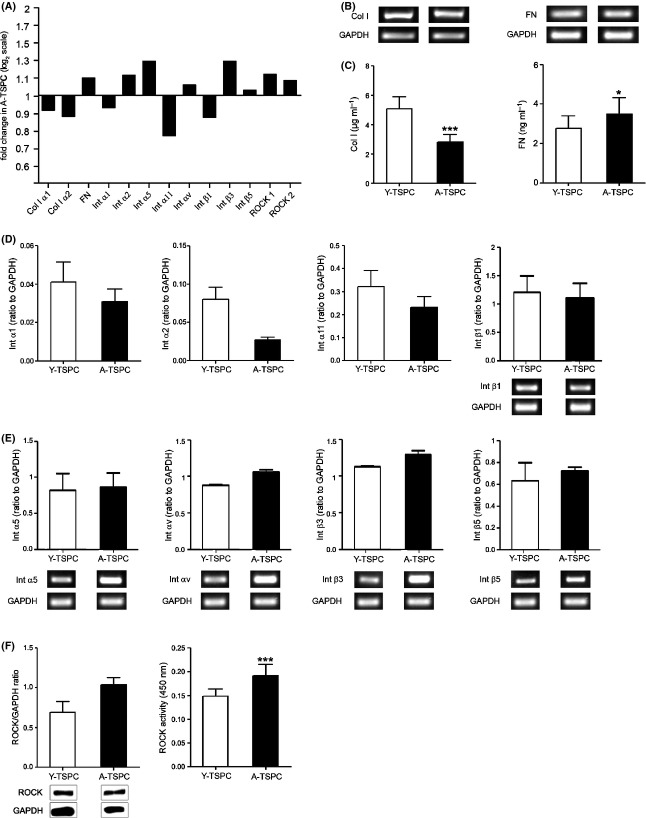
Validation of microarray results. (A) Fold changes in the expression of genes encoding for the extracellular matrix proteins collagen I (Col I) α1 and α2 and fibronectin (FN); collagen I-binding integrins (Int) α1, α2, α11, and β1; fibronectin-binding integrins α5, αv, β3, and β5; and ROCK1 and 2 kinase regulating actin stress fiber formation. The gene fold change is displayed as log_2_ ratio. Col I (left) and FN (right) expression on mRNA (B) and protein levels (C) analyzed by semi-quantitative PCR and ELISA, respectively. (D) Quantitative PCR for integrins α1, α2, and α11 and semi-quantitative PCR for the corresponding β1 integrin. (E) Densitometric quantification of semi-quantitative PCR for integrins α5, αV, β3, and β5. Representative PCR bands are shown beneath. (F) Western blot analysis of ROCK1 expression. (G) ELISA assay for ROCK2 activity. The above-mentioned analyses were reproduced at least twice independently with TSPC from three donors per group.

**Fig 6:**
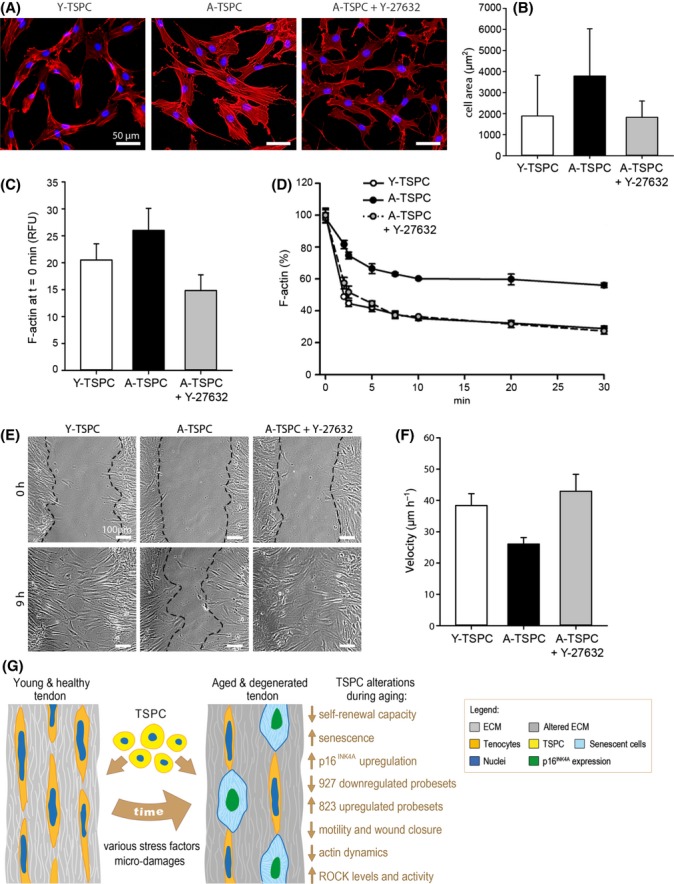
ROCK inhibition in A-TSPC. (A) Phalloidin staining of Y-TSPC, A-TSPC, and A-TSPC treated with the ROCK inhibitor 10 μm Y-27632. (B) Quantification of cell area. TSPC were cultivated on collagen I (200 cells per donor group were analyzed). (C) Comparison of F-actin content of Y-TSPC, A-TSPC, and A-TSPC + Y-27632 prior LanA treatment. RFU, relative fluorescent units. (D) Quantification of the F-actin amount upon LatA treatment. (E and F) Scratch assays on collagen I. Representative images at 0 and 9 h; cellular fronts are outlined with black lines. Cell velocity was calculated from four scratches per donor. The above-mentioned experiments were reproduced twice independently with three different donors per group. Bar charts show mean ± SD. (G) Schematic summary of the major findings detected in TSPC during tendon aging and degeneration. Young and healthy tendon tissue consists of abundant extracellular matrix, terminally differentiated tenocytes and resident stem and progenitor cells (TSPC). During aging, various stress factors and mechanical microdamages lead to altered tendon composition and functionality. Here, we report that in this process, TSPC accumulate several profound changes which altogether result in the exhaustion of their number and functional fitness.

The microarray data, the altered motility, and actin turnover of A-TSPC strongly suggested dysregulated cell–matrix interactions and actin stress fiber formation. Therefore, in the final part of the study, we compared the expression levels of several genes that play a pivotal role in the above-mentioned processes: (i) collagen I and fibronectin, which are essential for cell adhesion and migration; (ii) the collagen I-binding integrins α1β1, α2β1, and α11β1 and the fibronectin-binding α5β1, αvβ3, and αvβ5 (Docheva *et al*., 2007); and (iii) the ROCK kinase (ROCK1 and 2), which downstream signaling regulates actin stress fiber formation via myosin light chain (MLC) (Schmitz *et al*., 2000). The microarray data suggested that in A-TSPC, the collagen I and the corresponding integrins are downregulated, while the fibronectin, fibronectin-binding integrins, and ROCK1 and 2 are upregulated (Fig.5A and Table S6). ELISA assays demonstrated that the collagen I protein secretion is significantly reduced in A-TSPC, while fibronectin protein levels are increased (Fig.5C). PCR for collagen I- and fibronectin-binding integrins revealed that α1, α2, and α11 integrins are downregulated (Fig.5D) in contrast to integrins αv, β3, and β5, which are upregulated (Fig.5E). In the case of α2, we observed a discrepancy between the microarray and the PCR data, while for α5 and β1, the densitometric quantification of the PCR bands showed only slight differences; therefore, follow-up analyses on protein level will be required to conclude their expression changes. Next, in Fig5F and G, we compared the ROCK protein levels and activity in Y- and A-TSPC using Western blotting and ELISA assay. Our results clearly demonstrated augmented ROCK protein production and activity. Finally, we carried out inhibitory studies of ROCK to examine whether this will restore actin cytoskeleton dynamics and migration ability of aged TSPC. We treated A-TSPC with a well-characterized ROCK inhibitor Y-27632 (McMurray *et al*., 2011) and evaluated their cell area, F-actin organization and content, F-actin dynamics upon LatA treatment, and migration ability (Fig.6A–F). Our results demonstrated that upon inhibition of ROCK, A-TSPC re-establish a phenotype very similar to the Y-TSPC, thus confirming the central role of ROCK in TSPC aging. Taken together, our analyses suggest the dysregulation of cell–matrix interactions and ROCK kinase pathway to cause the observed functional deficit in A-TSPC motility and actin turnover.

## Discussion

The discovery of tendon stem/progenitor cells (TSPC) not only offered new insights into the biology of tendon cells but also demonstrated that distorted TSPC functions can be linked to the progression of tendon pathologies such as tendinous ossification (Bi *et al*., 2007). Here, we compared TSPC derived from young and healthy donors with those from aged and degenerative in order to identify key molecular and cellular players in tendon aging and degeneration. The major findings of our study demonstrated (i) profound TSPC self-renewal deficit accompanied with premature entry into cellular senescence; (ii) significant changes in the expression of genes regulating cell adhesion, migration, and cytoskeleton; and (iii) dysregulated cell–matrix interactions and actin dynamics, which we suggest to be dominant mechanisms leading to tendon aging and degeneration (Fig.6G).

We selected the Achilles tendon as a source for the TSPC isolation because of its clinical relevance and higher injury rate in elderly patients (Heckman *et al*., 2009). Due to patient morbidity and various background conditions, it was unattainable to obtain samples from aged and healthy individuals. Thus, the clinical samples used in our study did not allow the discrimination of tendon aging from age-related tendon degeneration. Follow-up studies should address this issue, as one prospect is to focus on tendon types, which can be obtained more easily from aged but healthy individuals. Furthermore, it remains to be answered whether stem/progenitor cells derived from ligaments are identical to TSPC and whether they behave similarly during ligament aging.

At the beginning of our investigation, we validated the TSPC character of the isolated cells by analyzing the expression of MSC- and tendon-related genes, and our results corroborate previous literature reporting on the identification of this cell type (Bi *et al*., 2007; Tempfer *et al*., 2009; Zhou *et al*., 2010; Mienaltowski *et al*., 2013). A classical feature of adult stem cells is their renewability and multilineage differentiation potential. Interestingly, a number of studies have demonstrated a direct link between the capacity of stem cells to proliferate and the onset of tissue aging (Finkel *et al*., 2007; Liu & Rando, 2011). In the MSC field, the following paradigm has been postulated: MSC derived from aged bone marrow form significantly less colonies, reach lower population doubling, and enter earlier into growth plateau (Baxter *et al*., 2004; Stolzing & Scutt, 2006; Stolzing *et al*., 2008; Kasper *et al*., 2009; Yu *et al*., 2011). An age-related decline in the growth kinetics was also reported for TSPC isolated from rat tendons; the study of Zhou *et al*. (2010) demonstrated significantly lower cell numbers, population doubling time, and clonogenicity of TSPC derived from old compared with young rats. In line with the above-mentioned literature, our study shows that human TSPC also exhibit a diminished self-renewal capacity, thus suggesting that the TSPC behave in accordance with the fundamental mechanism of age-associated depletion of the stem cell pool found in various adult stem cell niches. We propose that during tendon aging, the TSPC pool is being exhausted with respect to both sustainable and inducible self-renewability. This exhaustion is further amplified by activating cellular defense mechanisms such as entry into senescence. In the recent years, the elevation of tissue senescent cell numbers has been correlated with accumulative aging of the tissue. Furthermore, it has been shown that this process involves certain tumor suppressor pathways, namely p14^ARF^ and p16^INKA4^ (Sharpless & DePinho, 2007). For example, a study dealing with hematopoietic stem cells showed that the p16^INKA4^ mRNA levels are strongly upregulated in cells isolated from the bone marrow of aged mice (Janzen *et al*., 2006; Kasper *et al*., 2009). Similar findings were reported for MSC derived from aged rat and patient tissues (Kasper *et al*., 2009; Alt *et al*., 2012). Several *in vivo* models further emphasized the prominent role of p16^INKA4^ in tissue aging and even suggested that elimination or reduction in p16^INKA4^ expression can be one possible way to delay tissue aging and age-related diseases (Janzen *et al*., 2006; Baker *et al*., 2011). In p16^INKA4^ knockout mice, hematopoietic cells (Janzen *et al*., 2006) and neuronal progenitors (Molofsky *et al*., 2006) exhibit positively altered characteristics such as augmented self-renewal potential and proliferation rates along with reduced apoptotic cell numbers. Here, we found that TSPC derived from aged donors have higher senescence score and p16^INKA4^ levels than young TSPC, and we can suggest that targeting the mechanisms behind the p16^INKA4^ switch in aged tendon tissues might be a potential application to ameliorate tendon aging and degeneration.

Regarding the influence of aging on stem cells multipotency, there are very controversial results. Some studies showed that MSC from aged tissue samples exhibit a tendency to reduce the degree of three-lineage differentiation (Stolzing & Scutt, 2006; Yu *et al*., 2011), although a complete abolishment of lineage-specific commitment has not yet been reported. Others found no apparent differences in the extent of MSC differentiation during aging (Kasper *et al*., 2009; Katsara *et al*., 2011). Similar to the latter studies, we did not detect obvious changes in the multipotential capacity of aged versus young TSPC. This finding is slightly different to the observations of Zhou *et al*. (2010), who reported that TSPC from aged rats have unimpaired ability to form osteoblasts and chondrocytes, but form adipocytes more readily than young TSPC. This discrepancy can be due to variations in the methods used for isolating, culturing, and stimulating the cells as well as a species-related divergence. Hence, there are several remaining challenges to be addressed in the upcoming research (i) to standardize the protocols for TSPC enrichment, (ii) to establish efficient methods for terminal tenogenic differentiation *in vitro*, and (iii) to determine the TSPC self-renewal and differentiation capacity by serial transplantation assays *in vivo*.

To date, only few studies have analyzed the global gene expression patterns in somatic stem cells during aging. In general, they unveiled dysregulated gene expression related to cell cycle, stress response, inflammation, and genome integrity (Rossi *et al*., 2005; Chambers *et al*., 2007; Noda *et al*., 2009). With regard to tendons, our study is the first to report genome-wide microarray comparison between TSPC from young and aged donors. The gene ontology and literature annotation analysis suggested an intriguing transcriptomal shift in the expression of genes related to stem cell division and maintenance, cell–matrix and cell–cell interactions, cell migration, and actin cytoskeleton. Moreover, our microarray data are strongly supported by the enhanced actin stress fibers, lower actin turnover, and diminished migratory capacity registered in the aged TSPC. Interestingly, these findings are comparable to the study of Kasper *et al*. (2009), which evaluated the proteomes of MSC from young and aged mice and proposed that the main age-affected molecular factors are associated with cytoskeleton organization and oxidative stress defense. Age-related changes in the actin organization and migration potential have been also reported for keratinocytes and dermal fibroblasts (Reed *et al*., 2001; Ross *et al*., 2011). Actin cytoskeleton and its dynamics are pivotal for cell migration (Rottner & Stradal, 2011); therefore, our study suggests an ‘actin-centered’ problem in the aged TSPC, which can be strongly correlated with their migratory deficit as well as senescence uptake. Chen *et al*. (2000) have clearly demonstrated that senescent morphogenesis develops by a program involving activated p16^INKA4^/Rb pathway, enhancement of actin stress fiber formation, and redistribution of integrin focal adhesions. Additional evidence for our actin-cantered hypothesis is the detection of increased levels and activity of the ROCK kinases in the aged TSPC. ROCK proteins mediate the formation of RhoA-induced stress fibers through their effects on the phosphorylation of MLC and are involved in many aspects including cell morphology, mitosis, motility, and even differentiation (Schmitz *et al*., 2000). Upstream of the RhoA/ROCK signaling are the integrin receptors and their ECM ligands. Our expression analysis of collagen I, fibronectin, and their corresponding integrins showed that aged TSPC have a propensity to produce more fibronectin instead of collagen I, and concomitantly, a shift toward augmented expression of fibronectin-binding integrins was detected. In conclusion, we suggest that some of the mechanisms of aging such as replication, senescence, and stress response are inevitable to prevent tumor formation, but others may vary between cell types due to different niche environments, cell fates, and regulatory networks. Regarding TSPC, our study reports that dysregulated cell–matrix interactions and ROCK signaling might be key players in their aging process. Whether these gene dysregulations originate from an aged microenvironment or accumulated intrinsic cellular deficits has to be clarified in follow-up studies. Pursuing these questions can result in novel applications because previous studies have stated that the replicative senescence of keratinocytes *in vitro* can be evaded by an inhibition of ROCK (Chapman *et al*., 2010) and that senescent fibroblasts can be rescued by simply providing the cells with an ECM secreted by neonatal cells (Choi *et al*., 2011).

Taken together, we propose that the TSPC pool in the aged tendon tissue is exhausted in terms of size and functional fitness. Furthermore, the approach used in our study provides a novel platform for the identification of dominant molecular mechanisms that contribute to tendon aging and degeneration. An understanding of the mechanisms underlying these degenerative processes may lead to a major breakthrough in the prevention and treatment of tendon aging and age-associated complications.

## Experimental procedures

### Cell isolation and culture

We obtained cells from human nonruptured Achilles tendons from 16 patients, who had undergone diverse surgical operations in the Surgical Clinic of Ludwig Maximilians University in Munich (LMU). The procedure was approved by the Ethical Commission of the LMU Medical Faculty (grant No. 166-08), and informed consent was obtained from all donors. The samples were separated into two groups based on the clinical signs and pathological evaluation: young and healthy (abbreviated as Y-TSPC) and aged and degenerated (abbreviated as A-TSPC) (Table S1). The Y-TSPC group consisted of four donors with an average age of 28±5 years, while the A-TSPC group consisted of 12 donors with an average age of 63±14 years. One 37-year-old donor was included in this group because of clear clinical signs of tendon degeneration. TSPC were isolated according to Bi *et al*. (2007): the tendon tissue was minced into small pieces, enzymatically digested with 0.15% collagenase II (Worthington, Lakewood, NJ, USA) in culture medium at 37 °C overnight, filtered with sterile nylon mesh (100 μm pore size), and centrifuged at 500 g for 10 min. The cell pellet was resuspended in DMEM/Ham′s F-12 (1:1 mixture) supplemented with stabile glutamine, 1× MEM amino acids (all from Biochrom, Berlin, Germany), 10% FBS, and 1% L-ascorbic acid-2-phosphate (both from Sigma-Aldrich, Munich, Germany), and the TSPC were cultivated in a humidified incubator at constant 37 °C and 5% CO_2_. Based on the initial phenotypic analysis (monitoring primary cell yield, morphology, and expansion kinetics) of the A-TSPC cohort, which revealed very comparable morphological and cell growth characteristics, 3–4 TSPC strains were selected as representative and employed in all experiments. Unless otherwise stated in the figure legends, TSPC in passages 1–5 (PD 2–10) were used in the experiments.

### Flow cytometry (FACS) and immunocytochemistry

The protocols and antibodies used in the study are provided in the supporting information.

### Self-renewal analysis and WST-1 assay

Cumulative population doubling (PD) and colony-forming unit (CFU) assay were carried out as described in the study by Alberton *et al*. (2012) and the supporting information. For WST-1 assay, TSPC (3 × 10^3^ cells per cm^2^) were grown in 48- or 96-well plates for 24 h. Next, cells were incubated in complete culture medium containing 0.2% FBS, with or without FGF-2 (1 ng mL^−1^, PeproTech, Hamburg, Germany), or TGF-β_1_ (50 ng mL^−1^, R&D Systems, Wiesbaden, Germany) for 7 or 3 days, respectively. Thereafter, WST-1 reagent was applied according to the manufacturer's instructions (Roche, Penzberg, Germany). Optical density (OD) was measured at 420 nm and 620 nm using a Microtiter Reader (Thermo Scientific, Vantaa, Finland). The average OD value of Y-TSPC donors was set as control–100%.

### Senescence analysis

β-Galactosidase (β-gal) activity assay was performed using the Senescent Cells Staining Kit (Sigma-Aldrich). TSPC (1.5 × 10^3^ cells per cm^2^) from passages 4 (Y-TSPC PD 8; A-TSPC PD 7), 12 (Y-TSPC PD 25; A-TSPC PD 14), and 14 (Y-TSPC PD 28; A-TSPC PD 15) were grown in 12-well plates for 72 h, then fixed, and incubated with the kit's staining mixture for 16 h at 37 °C. Photographs were taken with AxioCamICc3 color camera on AxiovertS100 (Carl Zeiss, Oberkochen, Germany). In four random dish areas, β-gal-positive cells were expressed in percentage versus the total number of cells.

### Three-lineage differentiation

TSPC derived from three different donors per group were stimulated at passage 3 (PD 6) and 5 (Y-TSPC PD 10; A-TSPC PD 8) toward adipogenic, osteogenic, and chondrogenic lineages according to Alberton *et al*. (2012) and the supporting information.

### Genome-wide microarray and analysis

Comparison microarray and gene ontology analyses were performed as published by Schilling *et al*. (2008) and Rainer *et al*. (2006), respectively. Briefly, total RNA, isolated from TSPC of three young and three aged donors at passage 1 (PD 2), was hybridized onto Affymetrix GeneChips HG-U133 Plus2.0 (High Wycombe, UK) using the Affymetrix Expression Analysis Technical V2 manual. For details, refer to supporting information and Fig. S4, Table S4 and S5.

### Semi-quantitative and quantitative PCR

RNA isolation, cDNA synthesis, and PCR were performed as described in the study by Popov *et al*. (2011). For details of the procedures, primer sequences, and PCR conditions, refer to supporting information and Table S7.

### Migration experiments

An automated inverted microscope AxiovertS100 equipped with AxioCamMRm camera (Carl Zeiss) and biochamber (PeCon, Erbach, Germany) was used. For analysis of random migration, 1.5 × 10^3^ TSPC per cm^2^ were plated in 6-well dishes and incubated for 2 h prior imaging. Time-lapse was performed with 4 frames per h for 18 h. The image data were extracted with AxioVisionLE software (Carl Zeiss), and individual cell tracks were analyzed with ImageJ V1.38 software. For scratch assay, 1 × 10^4^ cells per cm^2^ were plated on collagen I (20 μg mL^−1^, Millipore, Billerica, MA, USA) or fibronectin (10 μg mL^−1^, Sigma-Aldrich)-coated 6-well dishes and were let to form confluent cell layers for 48 h. Prior imaging, the layers were scratched multiple times. Time-lapse was performed with 4 frames per h for 72 h. For each donor group, the initial length of four scratches and the time for complete scratch closure were measured and used for calculation of cell velocity.

### Quantification of cell area, F-actin staining, and latrunculin A treatment

TSPC from three different donors per group were cultivated on plastic dishes or collagen I-coated glass slides for 48–72 h. Cells were photographed, and cell areas were measured manually using the polygonal tool of the Image-Pro Plus V4 software (Media Cybernetics, Bathesda, MD, USA). For F-actin analysis, TSPC (5.5 × 10^3^ cells per cm^2^) were plated for 48 h on 96-well plates. Latrunculin A (LatA, Sigma-Aldrich) was diluted to 0.4 μm in complete culture medium containing 0.2% FBS and applied in a time-dependent manner. Afterward, the cells were fixed in 3.7% formalin/PHEM (6 mm PIPES, 25 mm HEPES, 10 mm EGTA, 3 mm MgCl_2_, pH 6.1) solution and permeabilized with 0.1% Triton X-100, and their F-actin was stained with phalloidin-AF546 (Invitrogen, Karlsruhe, Germany) as described in Docheva *et al*. (2010). DAPI dye was used for nuclear counterstaining. At each time point, TSPC were imaged with AxioCamMRm camera on AxiovertS100 microscope (Carl Zeiss). For quantification of the total F-actin amount, fluorescence signals were recorded at 573 nm using a fluorometer (Tecan, Crailsheim, Germany). In each donor group, the F-actin content at time point 0 was set to 100%.

### Western blotting and ELISA assays for collagen type I, fibronectin, and ROCK

Western blotting protocols are given in the supporting information. For ELISA assays, TSPC (8 × 10^3^ cells per cm) were seeded in 6-well dishes. After 3 days, the conditional medium, supplemented with protease inhibitors (Roche), was collected, and the cell number was counted. Secreted collagen type I was determined using Human Collagen I ELISA kit (Cosmo Bio, Tokyo, Japan) according to the manufacturer's instructions. OD was measured at 450 nm on a Microtiter Reader (Thermo Scientific). The collagen I concentration was calculated using collagen I standard curve and normalized to the cell number. The production of fibronectin was measured with Human Fibronectin ELISA kit (Millipore) following the manufacturer's recommendations and using 10 μg RIPA protein extracts from TSPC. The OD was measured at 450 nm, and the fibronectin concentration was interpolated from the kit's standard curve. ROCK2 activity was assessed by ROCK Activity kit (Cell Biolabs, San Diego, CA, USA). The protein input and OD readout are the same as in the fibronectin ELISA kit.

### Inhibition of ROCK in A-TSPC

A-TSPC were plated at different densities specific for each experimental set up and cultivated in basal media supplemented with 10 μM Y-27632 ROCK inhibitor (Sigma-Aldrich) for 72 h. Media was changed every day to ensure active ROCK inhibition. Treated A-TSPC were subjected to quantification of cell area, analyses of actin organization, content and turnover, and evaluation of migration ability as described above. Nontreated Y-TSPC and A-TSPC were used in parallel for comparison.

### Statistics

Quantitative data and statistical significance were analyzed using GraphPad Prism 5.0 software (GraphPad, La Jolla, CA, USA). Experiments were performed with minimum six different donors (three different donors per group). All quantitative data were acquired from two (*n* = 6) or three independent experiments (*n* = 9), each performed in duplicates or triplicates. Bar charts represent means and standard deviations. For testing statistical significance, unpaired Student's *t*-test was used. A *P*-value <0.05 was considered statistically significant and it was indicated in the figures as follows *P < 0.05, **P < 0.005, ***P < 0.0005.

## Supporting Information

**Fig. S1** Expression of stem cell markers and tendon-related genes in TSPC.

**Fig. S2** Colony-forming unit (CFU) assay conducted with three different TSPC densities.

**Fig. S3** Comparison of a three-lineage differentiation of Y-TSPC and A-TSPC.

**Fig. S4** Heatmap of the top 100 differentially expressed probesets.

**Table S1** Summary of the donor groups.

**Table S2** FACS analysis of MSC- and hematopoietic-related surface markers in TSPC.

**Table S3** Antibodies used in the study.

**Table S4** Complete microarray data.

**Table S5** Categorization of the 130 differentially expressed genes in the microarray.

**Table S6** Differentially expressed genes selected from the microarray data for further validation on RNA or protein level. **Table S7** Primer pairs and PCR conditions. Supplementary information of study protocols including detailed information on: Calculation of population doubling and colony-forming unit (CFU assay); FACS and immunocytochemistry protocols; Three-lineage differentiation protocols; RNA isolation, cDNA synthesis and PCR; Microarray analysis; Western blotting protocols.
