# Supplementary material for: Uncovering the cellular and molecular changes in tendon stem/progenitor cells attributed to tendon aging and degeneration
Source: Aging Cell. 2013 Jul 22;12(6):988–99. doi: 10.1111/acel.12124 (PMC4225469; doi:10.1111/acel.12124)
Supplement: Supplementary file 1 — Fig. S1 Expression of stem cell markers and tendon-related genes in TSPC. [file acel0012-0988-SD1.docx]

**Fig. S1.**

**
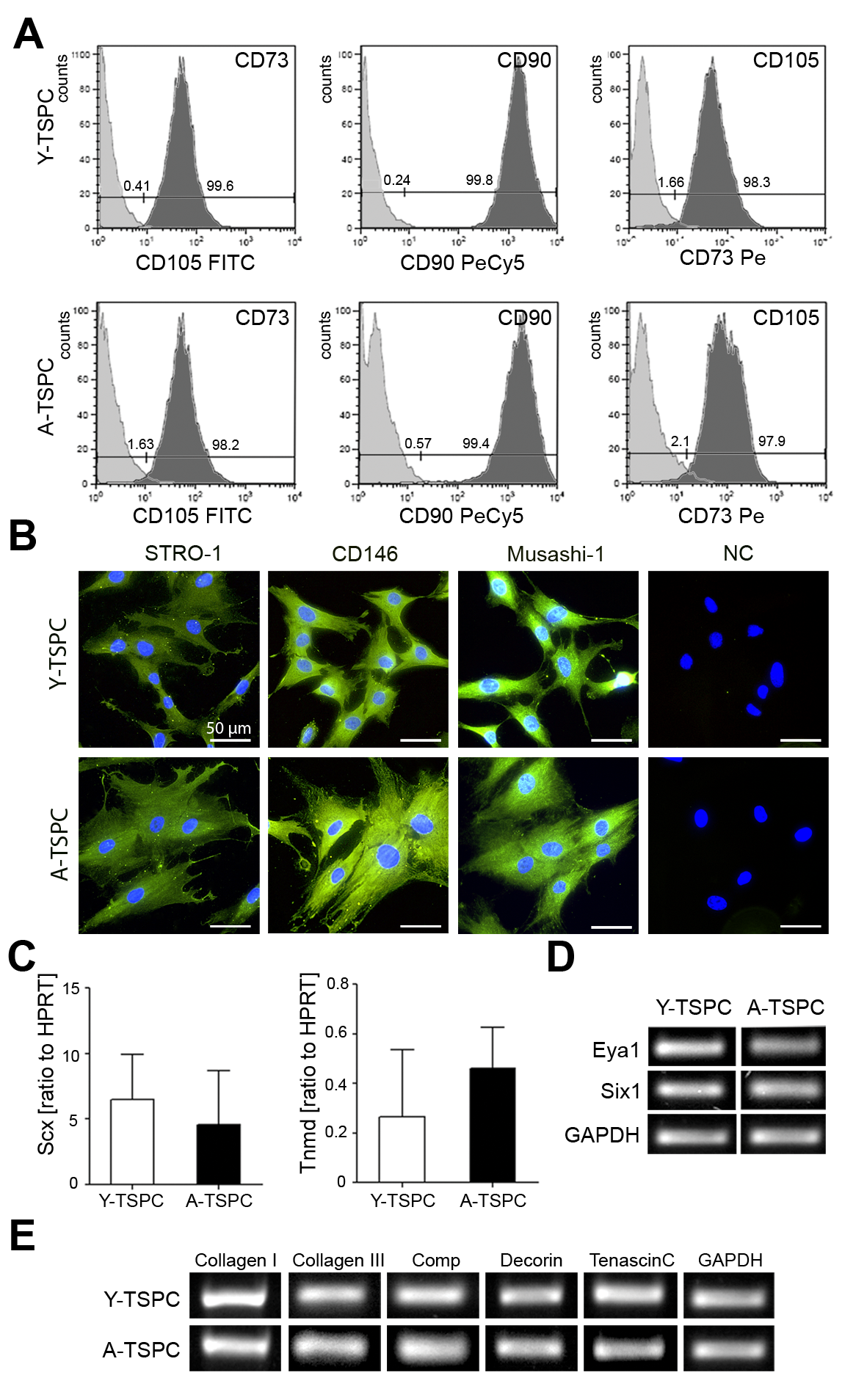
**

**Legends of supporting figures**

**Fig. S1:** Expression of stem cell- markers and tendon-related genes in TSPC. **(A)** FACS histograms for MSC-related surface antigens CD73, CD90 and CD105 expression in Y- and one A-TSPC. The data is representative for FACS analysis conducted with three different donors per group. **(B)** Immunocytochemistry for the stem cell markers STRO-1, CD146 and Musashi-1 (in green). Cell nuclei are labeled with DAPI (in blue). Representative images of two independent experiments with three different donors per group are shown. **(C)** Quantitative PCR analysis for Scleraxis (Scx) and tenomodulin (Tnmd). The data is shown as mean ± SD and represents two independent experiments with three donors per group. Semi-quantitative PCR analysis for the transcription factors Eya1 and Six1 **(D)** and extracellular matrix proteins (collagen type I and III, COMP, decorin, tenascin C) **(E)**. In C and D, representative images are shown as PCR was performed twice independently with three Y-TSPC and A-TSPC donors.
