# Supplementary material for: Uncovering the cellular and molecular changes in tendon stem/progenitor cells attributed to tendon aging and degeneration
Source: Aging Cell. 2013 Jul 22;12(6):988–99. doi: 10.1111/acel.12124 (PMC4225469; doi:10.1111/acel.12124)
Supplement: Supplementary file 2 — Fig. S2 Colony-forming unit (CFU) assay conducted with three different TSPC densities. [file acel0012-0988-SD2.docx]

**Fig. S2.**

**
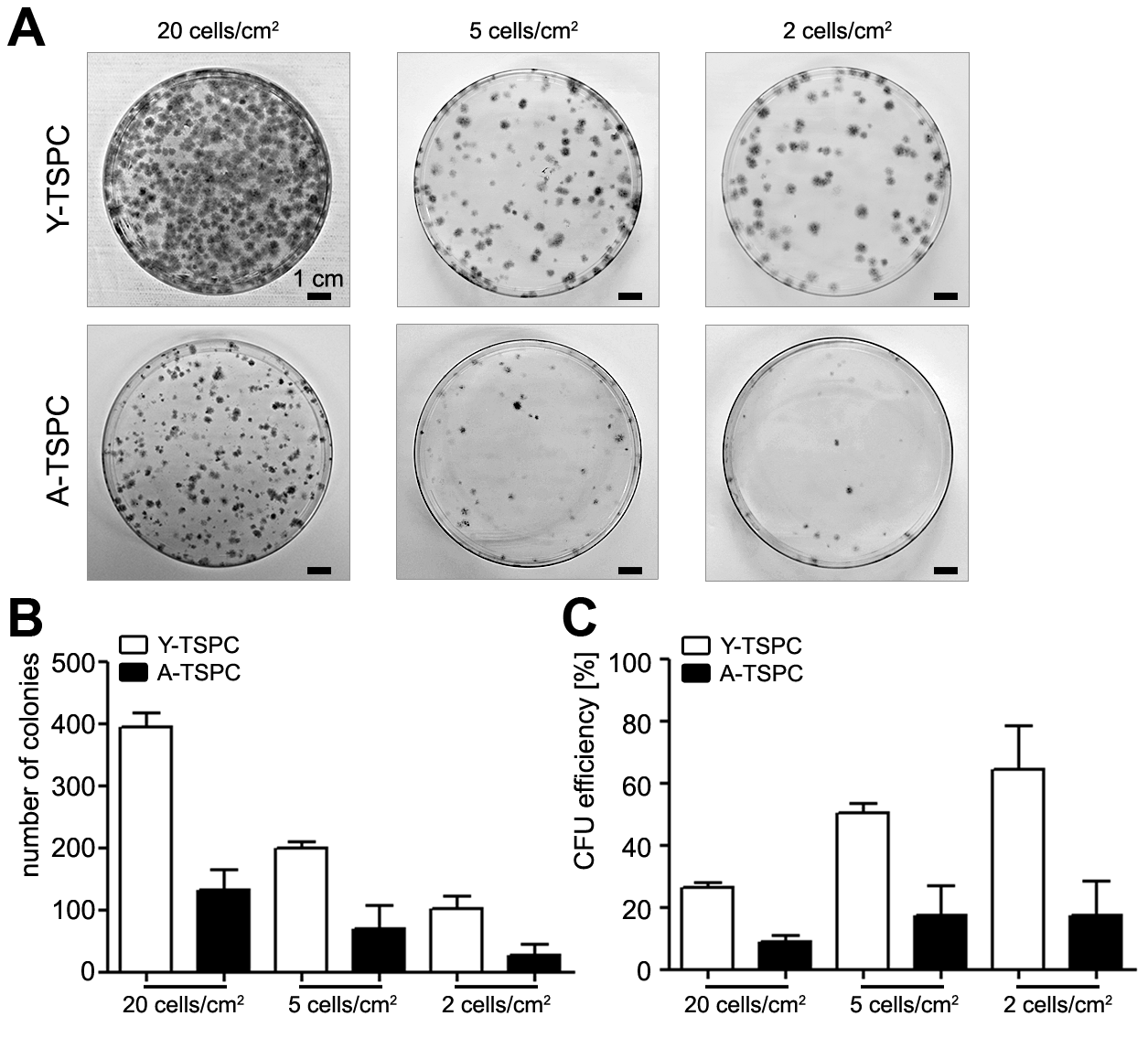
**

**Fig. S2:** Colony-forming unit (CFU) assay conducted with three different TSPC densities. **(A)** Crystal violet-stained colonies at day 14. **(B)** Number of formed colonies. **(C)** CFU efficiency. Bar charts show mean ± SD from two independent experiments with three donors per group.
