# Supplementary material for: Uncovering the cellular and molecular changes in tendon stem/progenitor cells attributed to tendon aging and degeneration
Source: Aging Cell. 2013 Jul 22;12(6):988–99. doi: 10.1111/acel.12124 (PMC4225469; doi:10.1111/acel.12124)
Supplement: Supplementary file 3 — Fig. S3 Comparison of a three-lineage differentiation of Y-TSPC and A-TSPC. [file acel0012-0988-SD3.docx]

**Fig. S3.**


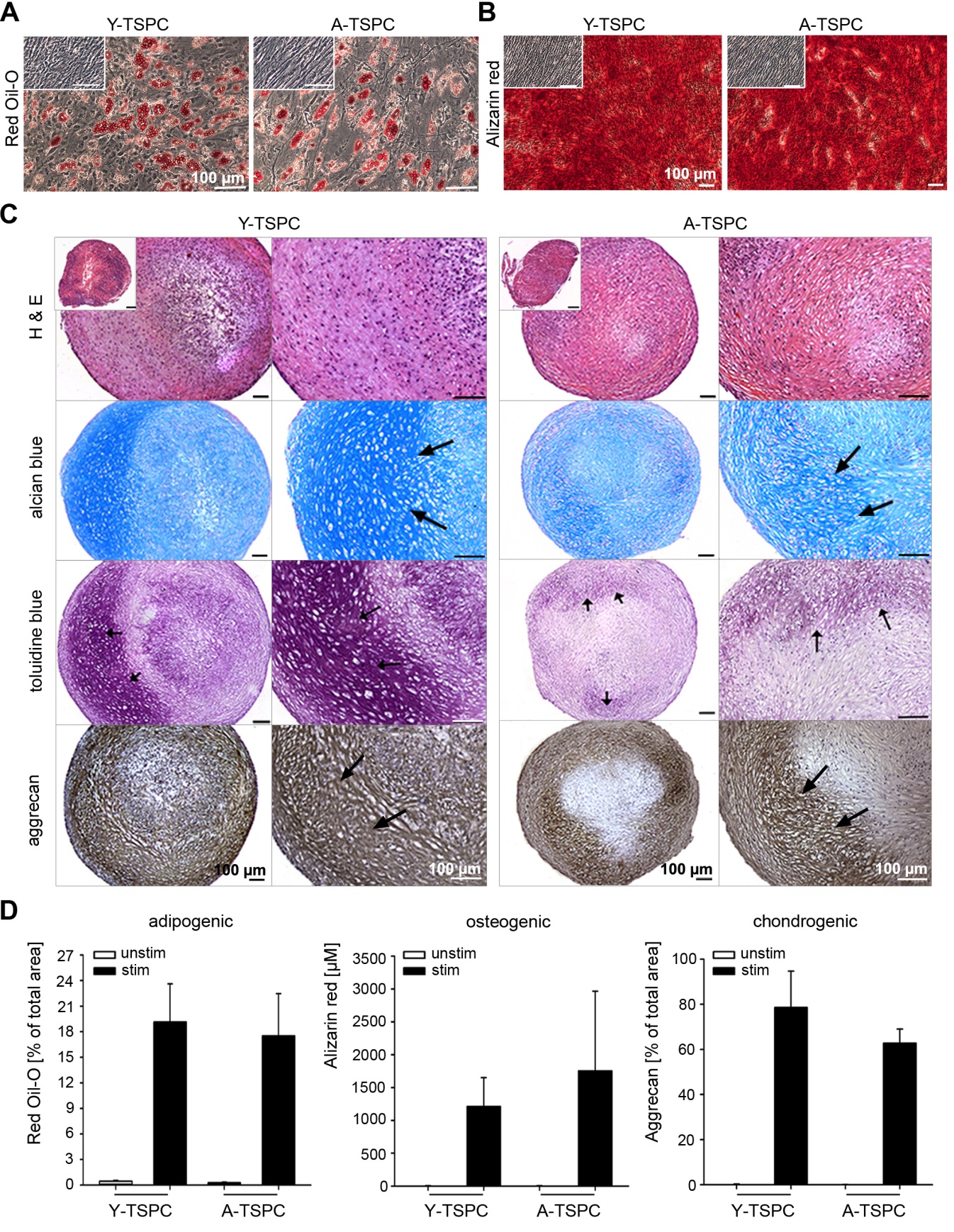


**Fig. S3:** Comparison of a three-lineage differentiation of Y-TSPC and A-TSPC. **(A)** Adipogenic differentiation. At day 21, formed lipid vacuoles were visualized by Oil Red-O staining. **(B)** Osteogenic differentiation. Alizarin Red staining for calcium depositions was performed at day 21 of stimulation. **(C)** Chondrogenic differentiation was performed for 35 day using pellet cultures. Tissue morphology and proteoglycan deposition was evaluated by haematoxylin & eosin (H&E), alcian blue and toluidine blue stainings as well as by aggrecan (cartilage-specific proteoglycan) immunostaining. Arrows indicate differentiated regions within the pellets. **(D)** Quantification of Oil Red-O staining, Alizarin Red accumulation and aggrecan-positive area. Differentiation protocols were repeated twice independently with three Y-TSPC and three A-TSPC donors; each donor in duplicates. Representative images for each staining are shown; all scale bars = 100 µm. Bar charts show mean ± SD from three different donors per group in two independent stimulations.
