# Supplementary material for: Uncovering the cellular and molecular changes in tendon stem/progenitor cells attributed to tendon aging and degeneration
Source: Aging Cell. 2013 Jul 22;12(6):988–99. doi: 10.1111/acel.12124 (PMC4225469; doi:10.1111/acel.12124)
Supplement: Supplementary file 4 — Fig. S4 Heatmap of the top 100 differentially expressed probesets. [file acel0012-0988-SD4.docx]

**Fig. S4.**

**
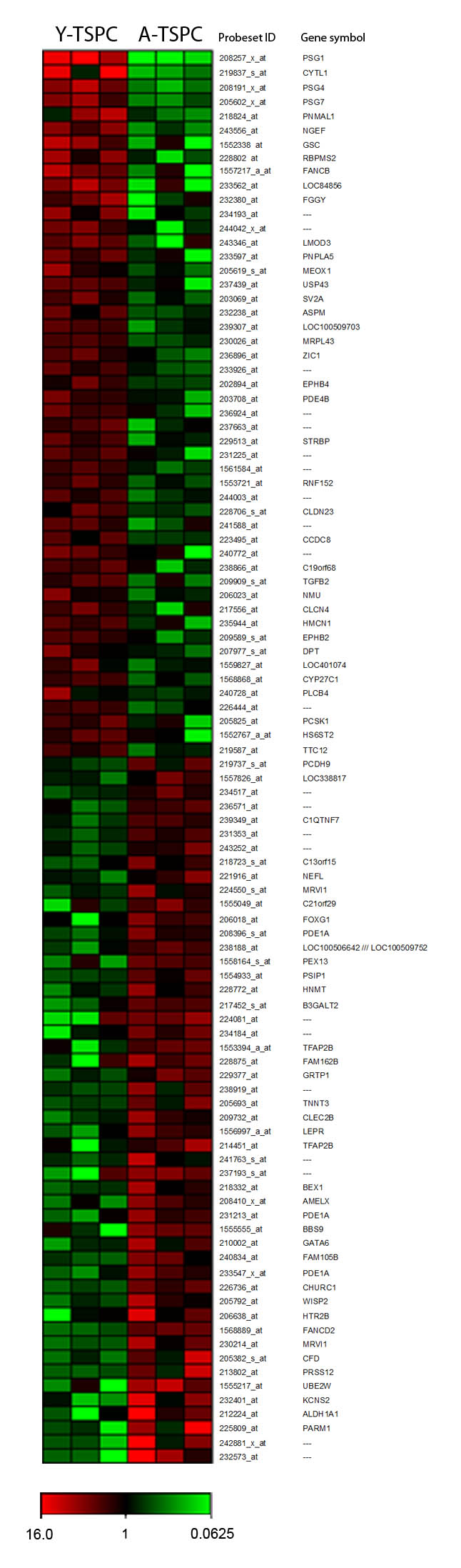
**

**Fig. S4**: Heatmap of the top 100 differentially expressed probesets. Color-coded microarray hybridization signals (red to green = high to low signals) of Y-TSPC and A-TSPC. The 100 depicted probesets showed at least 2 fold differential expression.
