# Supplementary material for: Uncovering the cellular and molecular changes in tendon stem/progenitor cells attributed to tendon aging and degeneration
Source: Aging Cell. 2013 Jul 22;12(6):988–99. doi: 10.1111/acel.12124 (PMC4225469; doi:10.1111/acel.12124)
Supplement: Supplementary file 5 — Table S1 Summary of the donor groups. [file acel0012-0988-SD5.docx]

**Table S1. Summary of the donor groups.**

| **Donor groups** | **Clinical indications** | **Histological examination**** | **Inclusion criteria** | **Exclusion Criteria** |
| --- | --- | --- | --- | --- |
| young/healthy  (N = 4; Mean age = 28±5) 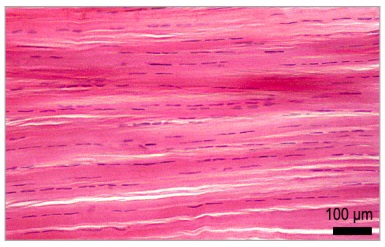 | - Accidents of the lower extremity with intact Achilles tendon;  - Surgical procedure:  Amputation | Degenerative lesions:  - Macroscopic findings (thicker, softer, yellowish, lost luster);  - Histological findings  1) structural anomalies: loss of normal pattern of collagen fiber alignment, areas of stromal homogenization due to hyalinisation;  2) loss of collagen stainability characterized by pale collagen fiber staining;  3) cellular changes:  rounded nuclei, increased cellularity, ingrowth of vessels. | - Donor consent;  - Surgical indications for lower extremity operation;  - Tissue not needed for further surgical procedures;  - Intact Achilles tendon;  - Males; | - Tendon rupture;  - Tendon abrasion;  - Chronic or local infections;  - Tendonitis;  - Tumor patients;  - Systemic or local glucocorticosteroid treatment;  - Autoimmune diseases (rheumatic diseases, collagenoses);  - Females (due to unknown hormonal effects) |
| aged/degenerated  (N = 12; Mean age = 63±14*)  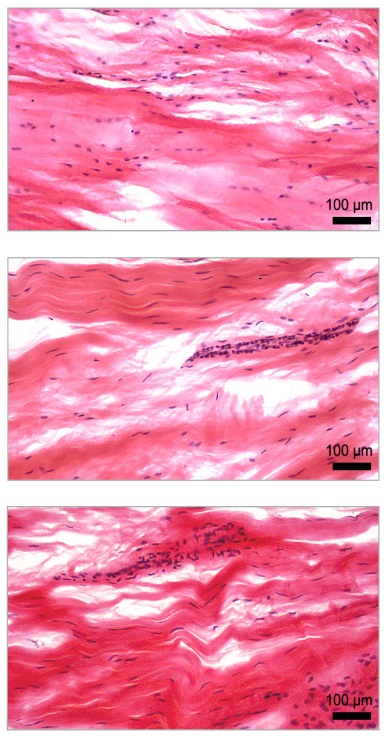 | - Deformities of ankle or mid-foot joints (osteoarthritis, arthropathy);  - Surgical procedure:  Arthrodesis with tenotomy of the Achilles tendon |  |  |  |

* One 37 years old donor was included in this group because of clear macroscopic and histological signs of Achilles tendon matrix degeneration.

** According to Puddu G, Ippolito E, Postacchini F (1976). A classification of achilles tendon disease. *Am J Sports Med.* **4**, 145-150.
