# Supplementary material for: Uncovering the cellular and molecular changes in tendon stem/progenitor cells attributed to tendon aging and degeneration
Source: Aging Cell. 2013 Jul 22;12(6):988–99. doi: 10.1111/acel.12124 (PMC4225469; doi:10.1111/acel.12124)
Supplement: Supplementary file 6 — Table S2 FACS analysis of MSC- and hematopoietic-related surface markers in TSPC. [file acel0012-0988-SD6.docx]

**Table S2. FACS analysis of MSC- and haematopoietic-related surface markers in TSPC.**

| **Surface antigens** | **Y-TSPC**  **[%]** | **A-TSPC**  **[%]** |
| --- | --- | --- |
| *Positive markers* | | |
| **CD73** | 99.2 ± 0.4 | 96.3 ± 3.1 |
| **CD90** | 99.5 ± 0.5 | 99.0 ± 1.0 |
| **CD105** | 98.7 ± 0.4 | 94.1 ± 6.9 |
| *Negative markers* | | |
| **CD19** | 0.55 ± 0.33 | 0.33 ± 0.06 |
| **CD34** | 4.10 ± 1.31 | 6.77 ± 1.99 |
| **CD45** | 0.40 ± 0.38 | 0.44 ± 0.18 |
| **HLA-DR** | 0.46 ± 0.26 | 0.34 ± 0.05 |

FACS analysis was performed with three different donors per group and mean values± standard deviation are shown in the table.
