# Supplementary material for: Uncovering the cellular and molecular changes in tendon stem/progenitor cells attributed to tendon aging and degeneration
Source: Aging Cell. 2013 Jul 22;12(6):988–99. doi: 10.1111/acel.12124 (PMC4225469; doi:10.1111/acel.12124)
Supplement: Supplementary file 7 — Table S3 Antibodies used in the study. [file acel0012-0988-SD7.docx]

**Table S3. Antibodies used in the study.**

| **Antibody** | **Antigen** | **Specificity** | **Company & Cat. number** | **Dilution** |
| --- | --- | --- | --- | --- |
| *FACS antibodies* | | | | |
| mouse anti-human CD19 - APC | CD19 (B4) | lymphocyte marker | Abcam  Ab18224 | 1:33 |
| mouse anti-human CD34 - APC | CD34 (gp105-120) | hematopoietic and vascular cell marker | Dianova  34A1-100T | 1:33 |
| mouse anti-human CD45 - APC | CD45 (B220; LCA; Ly5) | leukocyte marker | Abcam  Ab28106 | 1:33 |
| mouse anti-human CD73 - PE | CD73 (Ecto-5'-nucleotidase) | bone marrow MSC marker, lymphocyte subpopulations, follicular dendritic cells, epithelial and endothelial cells | BD Bioscience  550257 | 1:33 |
| mouse anti-human CD90 - PE-Cy5 | CD90 (Thy-1) | bone marrow MSC marker, hematopoietic cells and neurons | BD Bioscience  555597 | 1:33 |
| mouse anti-human CD105 - FITC | CD105 (endoglin) | bone marrow MSC marker, stromal and endothelial cells | BD Bioscience  561443 | 1:33 |
| mouse anti-human HLA-DR - APC | HLA-DR (MHC class II receptor) | macrophages, B-cells and dendritic cells | Beckman Coulter  IM3635 | 1:33 |
| mouse IgG1 isotype control – APC  (APC-IgG-ISO) |  | FACS isotype control antibody | Beckman Coulter  IM2475 | 1:33 |
| mouse IgG1 isotype control – FITC  (FITC-ISO) |  | FACS isotype control antibody | AbD Serotec  MCA1209F | 1:33 |
| mouse IgG1 κ isotype control - PE-Cy5  (PE-Cy5-ISO) |  | FACS isotype control antibody | BD Bioscience  555750 | 1:33 |
| mouse IgG1 κ isotype control – PE  (PE-IgG1-ISO) |  | FACS isotype control antibody | BD Bioscience  555749 | 1:33 |
| *Immunofluorescence antibodies* | | | | |
| rat anti-human CD44 | CD44 (H-CAM; Hermes; In-related) | hyaluronic acid receptor, widely expressed on most cell types including MSC | DSHB  Hermes-1 | 1:500 |
| rabbit anti-human  MCAM | CD146 (MCAM; A32; MUC18) | pericyte and MSC marker | Merck Millipore  04-1147 | 1:100 |
| rabbit anti-human Musashi-1 | Musashi-1 (Msi-1) | muscle, neural and pericyte stem cell marker | Merck Millipore  AB5977 | 1:250 |
| mouse anti-human p16 | p16^INK4A^ (CDKN2, INK4A, ARF) | cell cycle regulator | Santa Cruz  Sc-56330 | 1:50 |
| mouse anti-human STRO-1 | identity of the antigen is unknown | bone marrow MSC marker, erythroid precursors | R&D Systems  MAB1038 | 1:40 |
| goat anti-mouse AF488 |  | secondary antibody | Life Technologies  A11001 | 1:1000 |
| donkey anti-rabbit AF488 |  | secondary antibody | Life Technologies  A21206 | 1:500 |
| goat anti-rat AF546 |  | secondary antibody | Life Technologies  A11081 | 1:1000 |
| *Immunohistochemistry antibodies* | | | | |
| goat anti-human Aggrecan | Aggrecan (AGC1, CSPG1, MSK16) | cartilage-specific proteoglycan | R&D Systems  AF1220 | 1:400 |
| horse anti-goat biotinylated |  | secondary antibody | Vector Laboratories  BA-9500 | 1:400 |
| *Western blot antibodies* | | | | |
| goat anti-human ROCK-1 | ROCK-1 (p160ROCK) | downstream effector of Rho GTPase | Santa Cruz  Sc-6055 | 1:200 |
| rabbit anti-human GAPDH | GAPDH (GAPD) | housekeeping gene | Cell Signaling  2275-PC-100 | 1:2000 |
| donkey anti-goat HRP |  | secondary antibody | Santa Cruz  Sc-2020 | 1:4000 |
| goat anti-rabbit HRP |  | secondary antibody | Rockland  611-1322 | 1:4000 |

The alternative names of the antigens are limited to three synonyms.
