# Supplementary material for: Uncovering the cellular and molecular changes in tendon stem/progenitor cells attributed to tendon aging and degeneration
Source: Aging Cell. 2013 Jul 22;12(6):988–99. doi: 10.1111/acel.12124 (PMC4225469; doi:10.1111/acel.12124)
Supplement: Supplementary file 9 — Table S5 Categorization of the 130 differentially expressed genes in the microarray. [file acel0012-0988-SD9.docx]

**Table S5. Literature-base annotation of the 130 differentially expressed genes in the microarray.**

| **Gene symbol** | **Gene name** | **Fold change (Y/A)** | **Hits** | **“actin”** | **“cytoskeleton”** | **“migration”** | **“motility”** | **“cell adhesion”** | **“cell cell contact”** |
| --- | --- | --- | --- | --- | --- | --- | --- | --- | --- |
| **PSG1** | pregnancy specific beta-1-glycoprotein 1 | 57.233 | 1 |  |  |  | Lee et al. 1983 |  |  |
| **CYTL1** | cytokine-like 1 | 19.068 | 1 |  |  |  | Zou et al. 2008 |  |  |
| **PSG4** | pregnancy specific beta-1-glycoprotein 4 | 9.957 | 1 |  |  |  | Lee et al. 1983 |  |  |
| **PSG7** | pregnancy specific beta-1-glycoprotein 7 | 6.682 | 1 |  |  |  | Lee et al. 1983 |  |  |
| **NGEF** | neuronal guanine nucleotide exchange factor | 5.925 | 1 | Sahin et al. 2005  Winning et al. 2002  Shamah et al. 2001 |  |  |  |  |  |
| **GSC** | goosecoid homeobox | 5.887 | 5 | Cheng et al. 2011  Tabata et al. 2011  Cornell et al  1994  Sokol et al. 1992 | Cheng et al. 2011  Inaba et al. 2010  Stevens et al. 2007  Azuma et al. 2006  Deng et al. 1997 | Niehrs et al. 1993 | Niehrs et al. 1993 | Wacker et al. 1998  Montross et al. 2000  Al Saleh et al. 2011 |  |
| **LMOD3** | leiomodin 3 (fetal) | 3.43 | 1 |  |  |  |  |  | Campellone et al. 2010  De Minicis et al. 2007 |
| **MEOX1** | mesenchyme homeobox 1 | 3.255 | 1 | Gianakopoulos et al. 2005 |  |  |  |  |  |
| **SV2A** | synaptic vesicle glycoprotein 2A | 3.161 | 3 | Lai et al. 2001  Bridgman et al. 1999  Zimmermann 1996  Feany & Buckley, 1993 | Lai et al. 2001  Bridgman et al. 1999  Zimmermann 1996  Snow et al. 1996 |  | Feany & Buckley, 1993 |  |  |
| **ZIC1** | Zic family member 1 (odd-paired homolog, Drosophila) | 2.978 | 1 |  |  |  |  | Cornish et al. 2009 |  |
| **EPHB4** | EPH receptor B4 | 2.877 | 5 | Hugo et al. 2009  Truitt et al. 2010  Kimura et al. 2009  Taylor et al. 2007  Hamada et al. 2003 |  | Noren et al. 2009  Héroult et al. 2010  Astin et al. 2010  Chiu et al. 2009  Sakamoto et al. 2004  Stein et al. 1998 | Noren et al. 2009  Héroult et al. 2010  Astin et al. 2010  Chiu et al. 2009  Sakamoto et al. 2004  Stein et al. 1998 | Noren et al. 2009  Héroult et al. 2010  Astin et al. 2010  Chiu et al. 2009  Sakamoto et al. 2004  Stein et al. 1998 | Noren et al. 2009  Héroult et al. 2010  Astin et al. 2010  Chiu et al. 2009  Sakamoto et al. 2004  Stein et al. 1998 |
| **CLDN23** | claudin 23 | 2.75 | 1 |  |  |  |  | Tsukita & Furuse, 2000 |  |
| **NMU** | neuromedin U | 2.659 | 3 | Kojima et al. 2007  Yamanaka et al. 2002  Conde et al. 1998 | Shan et al. 2005 | Ketterer et al. 2009 |  |  |  |
| **PDE4B*** | phosphodiesterase 4B, cAMP-specific (phosphodiesterase E4 dunce homolog, Drosophila) | 2.637 | 4 | Landells et al. 2001 |  | García-Conesa et al. 2009  Sturz et al. 2004 | Binger et al. 2009 | Ariga et al. 2004 |  |
| **HMCN1** | hemicentin 1 | 2.604 | 1 |  |  |  |  |  | Xu & Vogel, 2011  Xu et al. 2007 |
| **EPHB2** | EPH receptor B2 | 2.589 | 3 |  |  | Arthur et al. 2011  He et al. 2010  Stimamiglio et al. 2010  Chumley et al. 2007  Nakada et al. 2004  Conover et al. 2000  Senior et al. 2010 |  | Arthur et al. 2011  Pfaff et al. 2008 | Stein et al. 1998 |
| **HS6ST2** | heparan sulfate 6-O-sulfotransferase 2 | 2.484 | 2 |  |  | Smeds et al. 2003 | Smeds et al. 2003 |  |  |
| **DPT*** | dermatopontin | 2.41 | 3 | Choi et al. 2009  Pavlik et al. 2008  Pavlik et al. 2007  Behnam et al. 2006  Hoeger et al. 1998  Villone et al. 19931. | Leterrier et al. 2009  Pavlik et al. 2008  Tatenhorst et al. 2004 |  |  |  | Kato et al. 2011  Okamoto et al. 2010  Cario-Toumaniantz et al. 2007  Behnam et al. 2006  Okamoto & Fujiwara, 2006  Takeda et al. 2002 |
| **CLCN4*** | chloride channel 4 | 2.379 | 1 |  |  | Ishiguro et al. 2010 |  |  |  |
| **LDB2** | LIM domain binding 2 | 2.353 | 4 | Umar et al. 2003  Kotaka et al. 2001 | Kotaka et al. 2000 | Storbeck et al. 2009 |  | Xu et al. 2007 |  |
| **SOX6*** | SRY (sex determining region Y)-box 6 | 2.343 | 2 | Dumitriu et al. 2006 |  |  |  | Hamada-Kanazawa et al. 2004  Pala et al. 2008 |  |
| **MDC1** | mediator of DNA-damage checkpoint 1 | 2.287 | 1 |  |  |  |  | Wilson et al. 2011 |  |
| **ACAP2** | ArfGAP with coiled-coil, ankyrin repeat and PH domains 2 | 2.254 | 2 | Furman et al. 2002  Jackson et al. 2000. |  |  |  | Furman et al. 2002  Ma et al. 2007  Zhang et al. 2009 |  |
| **COL11A1*** | collagen, type XI, alpha 1 | 2.247 | 1 |  |  |  |  | Zhao et al. 2009  Schmalbach et al. 2004 |  |
| **CHST1** | carbohydrate (keratan sulfate Gal-6) sulfotransferase 1 | 2.243 | 1 |  |  |  |  | Li et al. 2001  Li et al. 1999. |  |
| **SYK** | spleen tyrosine kinase | 2.218 | 4 |  |  | Neuhaus et al. 2011  Pearce et al. 2011  Christie et al. 2010  Schymeinsky et al. 2005  Schymeinsky et al. 2007  Lee et al. 2007  Lee et al. 2007  Inatome et al 2001 | Luangdilok et al. 2007  Zhang et al. 2009 | Cao et al. 2010  Mócsai et al. 2010  Buchner et al. 2010 | Buchner M et al. 2010  Dunant N et al. 1997 |
| **EPB41L4B** | erythrocyte membrane protein band 4.1 like 4B | 2.206 | 2 | McClatchey et al. 2009  Schulz et al. 2010  Lee et al. 2008  Schulz et al. 2007  Yu et al. 2011 |  |  |  | Yu et al. 2011  Yu et al. 2010 |  |
| **AMPH** | amphiphysin | 2.154 | 3 | Yamada et al. 2007  Kusumi et al. 2007  Usui et al. 2004 |  | Otsuka et al. 2003 |  | Otsuka et al. 2003  Messina et al. 2003  Zaidel-Bar et al. 2010 |  |
| **ZNF185** | zinc finger protein 185 (LIM domain) | 2.112 | 1 | Zhang et al. 2006 |  |  |  |  |  |
| **CPEB3** | cytoplasmic polyadenylation element binding protein 3 | 2.077 | 1 |  |  | Jones et al. 2008 |  |  |  |
| **ADM** | adrenomedullin | 0.498 | 3 |  |  | Chauhan et al. 2009  Xia et al. 2004  Vergaño-Vera et al. 2010  Chauhan et al. 2009  Xia et al. 2004  Fukai et al. 2003  Miyashita et al. 2003  Huang et al. 2004  Kohno et al. 1999  Horio et al. 1995  Kohno et al. 1997 | Chiu et al. 2010  Xia et al. 2004  Chauhan et al. 2009  Huang et al. 2004  Miyashita et al. 2003 | Jin et al. 2011  Kim et al. 2003  Hagi-Pavli et al. 2004  Hagi-Pavli et al. 2005  Ah Kioon et al. 2010 |  |
| **KIAA1772** | KIAA1772 | 0.463 | 1 |  |  | Nagase et al. 2000 |  |  |  |
| **ARID5B** | AT rich interactive domain 5B (MRF1-like) | 0.46 | 1 | Watanabe et al. 2002 |  |  |  |  |  |
| **EFHD1** | EF-hand domain family, member D1 | 0.459 | 1 |  | Dütting et al. 2011 |  |  |  |  |
| **FSTL3** | follistatin-like 3 (secreted glycoprotein) | 0.455 | 1 |  | Fuchshofer et al. 2009 |  |  |  |  |
| **PTGES*** | prostaglandin E synthase | 0.455 | 1 |  |  |  |  | Speirs et al. 2010 |  |
| **ANGPTL4*** | angiopoietin-like 4 | 0.448 | 4 |  | Cazes et al. 2006  Galaup et al. 2006  Ito et al. 2003 | Cazes et al. 2006  Galaup et al. 2006  Goh et al. 2010 | Cazes et al. 2006  Galaup et al. 2006  Goh et al. 2010 | Galaup et al. 2006  Goh et al. 2010  Chomel et al. 2009  Cazes et al. 2006 |  |
| **GSTM5** | glutathione S-transferase mu 5 | 0.436 | 1 |  | Eddy et al. 2003 |  |  |  |  |
| **GSTM1** | glutathione S-transferase mu 1 | 0.428 | 1 |  |  |  | Tirumala Vani et al. 2010 |  |  |
| **TACSTD2** | tumor-associated calcium signal transducer 2 | 0.427 | 2 |  |  | Villablanca et al. 2006 |  | Nakatsukasa et al. 2010 |  |
| **OLR1** | oxidized low density lipoprotein (lectin-like) receptor 1 | 0.409 | 1 |  |  |  |  | Sakamoto et al. 2009 |  |
| **IFITM1*** | interferon induced transmembrane protein 1 (9-27) | 0.401 | 2 |  |  | Yu et al. 2011 |  | Han et al. 2011  Tanaka et al. 2005 |  |
| **MYPN** | myopalladin | 0.399 | 1 |  |  | Otey et al. 2005 |  |  |  |
| **PCDH9*** | protocadherin 9 | 0.396 | 1 |  |  |  |  | Kim et al. 2010  Kim et al. 2011 |  |
| **HNMT*** | histamine N-methyltransferase | 0.374 | 1 | Otey et al. 2005  Bang et al. 2001. |  |  |  |  |  |
| **PDE1A*** | phosphodiesterase 1A, calmodulin-dependent | 0.359 | 3 | Zhou et al. 2010 |  |  | Tharayil et al. 2009 |  | Giachini et al. 2011  Zhou et al. 2010  Rybalkin et al. 2003 |
| **MRVI1*** | murine retrovirus integration site 1 homolog | 0.310 | 3 |  | Fabrizi et al. 2006  Tatenhorst et al. 2004 |  |  | Schinner et al. 2011 | Yaroslavskiy et al. 2010  Desch et al. 2010  Frei et al. 2009  Antl et al. 2007  Geiselhöringer et al. 2004  Fritsch et al. 2004 |
| **TFAP2B*** | transcription factor AP-2 beta (activating enhancer binding protein 2 beta) | 0.298 | 1 | Karagounis et al. 2010  Baltgalvis et al. 2009  Nakashima et al. 2007  Haddad et al. 2006  Mitch et al. 2005  Sacheck et al. 2004 |  |  |  |  |  |
| **CLEC2B** | C-type lectin domain family 2, member B | 0.29 | 3 | Pollitt et al. 2010 |  |  |  | Ozaki et al. 2009 | Watson et al. 2007  Kato et al. 2008  Pollitt et al. 2010  Chaipan et al. 2010 |
| **AMELX** | amelogenin (amelogenesis imperfecta 1, X-linked) | 0.276 | 2 | Sehic et al. 2010  Chen et al. 1992 |  | Hatakeyama et al. 2006 |  |  |  |
| **GATA6** | GATA binding protein 6 | 0.263 | 3 | Leppäranta et al. 2010  Wada et al. 2008  Zhang et al. 2007  Kanematsu et al. 2007  Yin et al. 2005  Kubo et al. 2004  Abe et al. 2003  Wada et al. 2002 |  |  | Fletcher et al. 2006 | Umetani et al. 2001 |  |
| **WISP2** | WNT1 inducible signaling pathway protein 2 | 0.245 | 2 |  |  | Banerjee et al. 2008 | Banerjee et al. 2008  Phillips et al. 2008  Lake et al. 2003 |  |  |
| **ALDH1A1** | aldehyde dehydrogenase 1 family, member A1 | 0.131 | 2 |  | Walsh et al. 2009  Simmen et al. 2008 |  | Moreb et al. 2008 |  |  |

The 130 differentially expressed genes (fold changes lower than 0.5 and greater than 2) were assigned to NCBI PubMed literature search (http://www.ncbi.nlm.nih.gov/pubmed) and were categorized in the following six categories: i) cell-cell contact; ii) cell adhesion; iii) motility; iv) migration; v) cytoskeleton and vi) actin-related genes. Each category was calculated in percentage from 130 genes (see Fig. 3D). Genes that produced literature hits in at least one of the categories are included in the table (n = 53, 40.8%). Genes marked with asterisk were present in the microarray with double or triple probesets.

**References**

Abe M, Hasegawa K, Wada H, Morimoto T, Yanazume T, Kawamura T, Hirai M, Furukawa Y, Kita T. GATA-6 is involved in PPARgamma-mediated activation of differentiated phenotype in human vascular smooth muscle cells. Arterioscler Thromb Vasc Biol. 2003 Mar 1;23(3):404-10. Epub 2003 Jan 30. PubMed PMID: 12615657.

Ah Kioon MD, Asensio C, Ea HK, Uzan B, Cohen-Solal M, Lioté F. Adrenomedullin increases fibroblast-like synoviocyte adhesion to extracellular matrix proteins by upregulating integrin activation. Arthritis Res Ther. 2010;12(5):R190.

Al Saleh S, Sharaf LH, Luqmani YA. Signalling pathways involved in endocrine resistance in breast cancer and associations with epithelial to mesenchymal transition (Review). Int J Oncol. 2011 May;38(5):1197-217.

Antl M, von Brühl ML, Eiglsperger C, Werner M, Konrad I, Kocher T, Wilm M, Hofmann F, Massberg S, Schlossmann J. IRAG mediates NO/cGMP-dependent inhibition of platelet aggregation and thrombus formation. Blood. 2007 Jan 15;109(2):552-9.

Ariga M, Neitzert B, Nakae S, Mottin G, Bertrand C, Pruniaux MP, Jin SL, Conti M. Nonredundant function of phosphodiesterases 4D and 4B in neutrophil recruitment to the site of inflammation. J Immunol. 2004 Dec 15;173(12):7531-8.

Arthur A, Zannettino A, Panagopoulos R, Koblar SA, Sims NA, Stylianou C, Matsuo K, Gronthos S. EphB/ephrin-B interactions mediate human MSC attachment, migration and osteochondral differentiation. Bone. 2011 Mar 1;48(3):533-42. Epub 2010 Nov 5. PubMed PMID: 21056708.

Astin JW, Batson J, Kadir S, Charlet J, Persad RA, Gillatt D, Oxley JD, Nobes CD. Competition amongst Eph receptors regulates contact inhibition of locomotion and invasiveness in prostate cancer cells. Nat Cell Biol. 2010 Dec;12(12):1194-204.

Azuma R, Kitagawa T, Kobayashi H, Konagaya A. Particle simulation approach for subcellular dynamics and interactions of biological molecules. BMC Bioinformatics. 2006 Dec 12;7 Suppl 4:S20. PubMed PMID: 17217513; PubMed Central PMCID: PMC1780110.

Baltgalvis KA, Berger FG, Peña MM, Davis JM, White JP, Carson JA. Muscle wasting and interleukin-6-induced atrogin-I expression in the cachectic Apc (Min/+) mouse. Pflugers Arch. 2009 Mar;457(5):989-1001. Epub 2008 Aug 19. PubMed PMID: 18712412; PubMed Central PMCID: PMC2867110.

Banerjee S, Dhar G, Haque I, Kambhampati S, Mehta S, Sengupta K, Tawfik O, Yeger H, Perbal B. The CCN family of genes: a perspective on CCN biology and therapeutic potential. J Cell Commun Signal. 2007 Dec;1(3-4):159-64. Epub 2008 Jun 21. PubMed PMID: 18568428; PubMed Central PMCID: PMC2443235.

Bang ML, Mudry RE, McElhinny AS, Trombitás K, Geach AJ, Yamasaki R, Sorimachi H, Granzier H, Gregorio CC, Labeit S. Myopalladin, a novel 145-kilodalton sarcomeric protein with multiple roles in Z-disc and I-band protein assemblies. J Cell Biol. 2001 Apr 16;153(2):413-27. PubMed PMID: 11309420; PubMed Central PMCID: PMC2169455.

Behnam K, Murray SS, Brochmann EJ. BMP stimulation of alkaline phosphatase activity in pluripotent mouse C2C12 cells is inhibited by dermatopontin, one of the most abundant low molecular weight proteins in demineralized bone matrix. Connect Tissue Res. 2006;47(5):271-7.

Binger T, Stich S, Andreas K, Kaps C, Sezer O, Notter M, Sittinger M, Ringe J. Migration potential and gene expression profile of human mesenchymal stem cells induced by CCL25. Exp Cell Res. 2009 May 1;315(8):1468-79. Epub 2009 Jan 8. PubMed PMID: 19168060.

Bridgman PC. Myosin Va movements in normal and dilute-lethal axons provide support for a dual filament motor complex. J Cell Biol. 1999 Sep 6;146(5):1045-60. PubMed PMID: 10477758; PubMed Central PMCID: PMC2169472.

Buchner M, Baer C, Prinz G, Dierks C, Burger M, Zenz T, Stilgenbauer S, Jumaa H, Veelken H, Zirlik K. Spleen tyrosine kinase inhibition prevents chemokine- and integrin-mediated stromal protective effects in chronic lymphocytic leukemia. Blood. 2010 Jun 3;115(22):4497-506.

Buchner M, Baer C, Prinz G, Dierks C, Burger M, Zenz T, Stilgenbauer S, Jumaa H, Veelken H, Zirlik K. Spleen tyrosine kinase inhibition prevents chemokine- and integrin-mediated stromal protective effects in chronic lymphocytic leukemia. Blood. 2010 Jun 3;115(22):4497-506.

Campellone KG, Welch MD. A nucleator arms race: cellular control of actin assembly. Nat Rev Mol Cell Biol. 2010 Apr;11(4):237-51. Epub 2010 Mar 18.

Cao L, Li L, Yang H, Yin H. Overexpression of P-selectin glycoprotein ligand-1 enhances adhesive properties of endothelial progenitor cells through Syk activation. Acta Biochim Biophys Sin (Shanghai). 2010 Aug;42(8):507-14. Epub 2010

Cario-Toumaniantz C, Boularan C, Schurgers LJ, Heymann MF, Le Cunff M, Léger J, Loirand G, Pacaud P. Identification of differentially expressed genes in human varicose veins: involvement of matrix gla protein in extracellular matrix remodeling. J Vasc Res. 2007;44(6):444-59.

Cazes A, Galaup A, Chomel C, Bignon M, Bréchot N, Le Jan S, Weber H, Corvol P, Muller L, Germain S, Monnot C. Extracellular matrix-bound angiopoietin-like 4 inhibits endothelial cell adhesion, migration, and sprouting and alters actin cytoskeleton. Circ Res. 2006 Nov 24;99(11):1207-15.

Chaipan C, Steffen I, Tsegaye TS, Bertram S, Glowacka I, Kato Y, Schmökel J, Münch J, Simmons G, Gerardy-Schahn R, Pöhlmann S. Incorporation of podoplanin into HIV released from HEK-293T cells, but not PBMC, is required for efficient binding to the attachment factor CLEC-2. Retrovirology. 2010 May 19;7:47.

Chauhan M, Yallampalli U, Dong YL, Hankins GD, Yallampalli C. Expression of adrenomedullin 2 (ADM2)/intermedin (IMD) in human placenta: role in trophoblast invasion and migration. Biol Reprod. 2009 Oct;81(4):777-83. Epub 2009 Jun 17. PubMed PMID: 19535789.

Chen LS, Couwenhoven RI, Hsu D, Luo W, Snead ML. Maintenance of amelogenin gene expression by transformed epithelial cells of mouse enamel organ. Arch Oral Biol. 1992 Oct;37(10):771-8. PubMed PMID: 1444889.

Cheng J, Tiyaboonchai A, Yamashita YM, Hunt AJ. Asymmetric division of cyst stem cells in Drosophila testis is ensured by anaphase spindle repositioning. Development. 2011 Mar;138(5):831-7. PubMed PMID: 21303845; PubMed Central PMCID: PMC3035088.

Chiu ST, Chang KJ, Ting CH, Shen HC, Li H, Hsieh FJ. Over-expression of EphB3 enhances cell-cell contacts and suppresses tumor growth in HT-29 human colon cancer cells. Carcinogenesis. 2009 Sep;30(9):1475-86.

Choi Y, Kim HP, Hong SM, Ryu JY, Han SJ, Song R. In situ visualization of gene expression using polymer-coated quantum-dot-DNA conjugates. Small. 2009 Sep;5(18):2085-91. PubMed PMID: 19517489.

Chomel C, Cazes A, Faye C, Bignon M, Gomez E, Ardidie-Robouant C, Barret A, Ricard-Blum S, Muller L, Germain S, Monnot C. Interaction of the coiled-coil domain with glycosaminoglycans protects angiopoietin-like 4 from proteolysis and regulates its antiangiogenic activity. FASEB J. 2009 Mar;23(3):940-9.

Christie TL, Carter A, Rollins EL, Childs SJ. Syk and Zap-70 function redundantly to promote angioblast migration. Dev Biol. 2010 Apr 1;340(1):22-9.Epub 2010 Jan 22. PubMed PMID: 20096681.

Chumley MJ, Catchpole T, Silvany RE, Kernie SG, Henkemeyer M. EphB receptors regulate stem/progenitor cell proliferation, migration, and polarity during hippocampal neurogenesis. J Neurosci. 2007 Dec 5;27(49):13481-90. PubMed PMID: 18057206. ;

Conde B, Sinués E, Gascon A, Alcala A, Lorenzo HC, Ruidiaz M. A new breast cancer cell line of epithelial origin is tumorigenic in athymic mice. Anticancer Res. 1998 May-Jun;18(3A):1751-5. PubMed PMID: 9673400.

Conover JC, Doetsch F, Garcia-Verdugo JM, Gale NW, Yancopoulos GD, Alvarez-Buylla A. Disruption of Eph/ephrin signaling affects migration and proliferation in the adult subventricular zone. Nat Neurosci. 2000 Nov;3(11):1091-7. PubMed PMID: 11036265.

Cornell RA, Kimelman D. Activin-mediated mesoderm induction requires FGF. Development. 1994 Feb;120(2):453-62. PubMed PMID: 8149920.

Cornish EJ, Hassan SM, Martin JD, Li S, Merzdorf CS. A microarray screen for direct targets of Zic1 identifies an aquaporin gene, aqp-3b, expressed in the neural folds. Dev Dyn. 2009 May;238(5):1179-94.

De Minicis S, Seki E, Uchinami H, Kluwe J, Zhang Y, Brenner DA, Schwabe RF. Gene expression profiles during hepatic stellate cell activation in culture and in vivo. Gastroenterology. 2007 May;132(5):1937-46.

Deng W, Lin H. Spectrosomes and fusomes anchor mitotic spindles during asymmetric germ cell divisions and facilitate the formation of a polarized microtubule array for oocyte specification in Drosophila. Dev Biol. 1997 Sep 1;189(1):79-94. PubMed PMID: 9281339.

Desch M, Sigl K, Hieke B, Salb K, Kees F, Bernhard D, Jochim A, Spiessberger B, Höcherl K, Feil R, Feil S, Lukowski R, Wegener JW, Hofmann F, Schlossmann J. IRAG determines nitric oxide- and atrial natriuretic peptide-mediated smooth muscle relaxation. Cardiovasc Res. 2010 Jun 1;86(3):496-505.

Dumitriu B, Patrick MR, Petschek JP, Cherukuri S, Klingmuller U, Fox PL, Lefebvre V. Sox6 cell-autonomously stimulates erythroid cell survival, proliferation, and terminal maturation and is thereby an important enhancer of definitive erythropoiesis during mouse development. Blood. 2006 Aug 15;108(4):1198-207. Epub 2006 Apr 20.

Dunant N, Ballmer-Hofer K. Signalling by Src family kinases: lessons learnt from DNA tumour viruses. Cell Signal. 1997 Sep;9(6):385-93. Review.

Dütting S, Brachs S, Mielenz D. Fraternal twins: Swiprosin-1/EFhd2 and Swiprosin-2/EFhd1, two homologous EF-hand containing calcium binding adaptor proteins with distinct functions. Cell Commun Signal. 2011 Jan 18;9:2.

Eddy EM, Toshimori K, O'Brien DA. Fibrous sheath of mammalian spermatozoa. Microsc Res Tech. 2003 May 1;61(1):103-15.

Fabrizi GM, Cavallaro T, Angiari C, Cabrini I, Taioli F, Malerba G, Bertolasi L, Rizzuto N. Charcot-Marie-Tooth disease type 2E, a disorder of thecytoskeleton. Brain. 2007 Feb;130(Pt 2):394-403. Epub 2006 Oct 18. PubMed PMID: 17052987.

Feany MB, Buckley KM. The synaptic vesicle protein synaptotagmin promotes formation of filopodia in fibroblasts. Nature. 1993 Aug 5;364(6437):537-40 PubMed PMID: 8166886.

Frei E, Huster M, Smital P, Schlossmann J, Hofmann F, Wegener JW. Calcium-dependent and calcium-independent inhibition of contraction by cGMP/cGKI in intestinal smooth muscle. Am J Physiol Gastrointest Liver Physiol. 2009 Oct;297(4):G834-9.

Fritsch RM, Saur D, Kurjak M, Oesterle D, Schlossmann J, Geiselhöringer A, Hofmann F, Allescher HD. InsP3R-associated cGMP kinase substrate (IRAG) is essential for nitric oxide-induced inhibition of calcium signaling in human colonic smooth muscle. J Biol Chem. 2004 Mar 26;279(13):12551-9.

Fuchshofer R, Stephan DA, Russell P, Tamm ER. Gene expression profiling of TGFbeta2- and/or BMP7-treated trabecular meshwork cells: Identification of Smad7 as a critical inhibitor of TGF-beta2 signaling. Exp Eye Res. 2009 Jun;88(6):1020-32. Epub 2009 Jan 18.

Fukai N, Shichiri M, Ozawa N, Matsushita M, Hirata Y. Coexpression of calcitonin receptor-like receptor and receptor activity-modifying protein 2 or 3 mediates the antimigratory effect of adrenomedullin. Endocrinology. 2003 Feb;144(2):447-53. PubMed PMID: 12538603.

Furman C, Short SM, Subramanian RR, Zetter BR, Roberts TM. DEF-1/ASAP1 is a GTPase-activating protein (GAP) for ARF1 that enhances cell motility through a GAP-dependent mechanism. J Biol Chem. 2002 Mar 8;277(10):7962-9. Epub 2001 Dec 31.

Galaup A, Cazes A, Le Jan S, Philippe J, Connault E, Le Coz E, Mekid H, Mir LM, Opolon P, Corvol P, Monnot C, Germain S. Angiopoietin-like 4 prevents metastasis through inhibition of vascular permeability and tumor cell motility and invasiveness. Proc Natl Acad Sci U S A. 2006 Dec 5;103(49):18721-6. Epub 2006 Nov 27. PubMed PMID: 17130448; PubMed Central PMCID: PMC1693729.

García-Conesa MT, Tribolo S, Guyot S, Tomás-Barberán FA, Kroon PA. Oligomeric procyanidins inhibit cell migration and modulate the expression of migration and proliferation associated genes in human umbilical vascular endothelial cells. Mol Nutr Food Res. 2009 Feb;53(2):266-76. PubMed PMID: 18979505. ; Sturz A, Bader B, Thierauch KH, Glienke J. EphB4 signaling is capable ofmediating ephrinB2-induced inhibition of cell migration. Biochem Biophys Res Commun. 2004 Jan 2;313(1):80-8. PubMed PMID: 14672701.

Geiselhöringer A, Werner M, Sigl K, Smital P, Wörner R, Acheo L, Stieber J, Weinmeister P, Feil R, Feil S, Wegener J, Hofmann F, Schlossmann J. IRAG is essential for relaxation of receptor-triggered smooth muscle contraction by cGMP kinase. EMBO J. 2004 Oct 27;23(21):4222-31.

Giachini FR, Lima VV, Carneiro FS, Tostes RC, Webb RC. Decreased cGMP level contributes to increased contraction in arteries from hypertensive rats: role of phosphodiesterase 1. Hypertension. 2011 Mar;57(3):655-63. Epub 2011 Jan 31.

Gianakopoulos PJ, Skerjanc IS. Hedgehog signaling induces cardiomyogenesis in P19 cells. J Biol Chem. 2005 Jun 3;280(22):21022-8. Epub 2005 Mar 26.

Goh YY, Pal M, Chong HC, Zhu P, Tan MJ, Punugu L, Lam CR, Yau YH, Tan CK, Huang RL, Tan SM, Tang MB, Ding JL, Kersten S, Tan NS. Angiopoietin-like 4 interacts with integrins beta1 and beta5 to modulate keratinocyte migration. Am J Pathol. 2010 Dec;177(6):2791-803. Epub 2010 Oct

Goh YY, Pal M, Chong HC, Zhu P, Tan MJ, Punugu L, Tan CK, Huang RL, Sze SK, Tang MB, Ding JL, Kersten S, Tan NS. Angiopoietin-like 4 interacts with matrix proteins to modulate wound healing. J Biol Chem. 2010 Oct 22;285(43):32999-3009.

Haddad F, Adams GR, Bodell PW, Baldwin KM. Isometric resistance exercise fails to counteract skeletal muscle atrophy processes during the initial stages of unloading. J Appl Physiol. 2006 Feb;100(2):433-41. Epub 2005 Oct 20. PubMed PMID: 16239603.

Hagi-Pavli E, Farthing PM, Henshaw FN, Kapas S. Presentation of ICAM-1 protein at the cell surface of oral keratinocytes in the presence of adrenomedullin and corticotrophin. Cell Physiol Biochem. 2005;15(1-4):167-74.

Hagi-Pavli E, Farthing PM, Kapas S. Stimulation of adhesion molecule expression in human endothelial cells (HUVEC) by adrenomedullin andcorticotrophin. Am J Physiol Cell Physiol. 2004 Feb;286(2):C239-46.

Hamada K, Oike Y, Ito Y, Maekawa H, Miyata K, Shimomura T, Suda T. Distinct roles of ephrin-B2 forward and EphB4 reverse signaling in endothelial cells. Arterioscler Thromb Vasc Biol. 2003 Feb 1;23(2):190-7. PubMed PMID: 12588758.

Hamada-Kanazawa M, Ishikawa K, Nomoto K, Uozumi T, Kawai Y, Narahara M, Miyake M. Sox6 overexpression causes cellular aggregation and the neuronal differentiation of P19 embryonic carcinoma cells in the absence of retinoic acid. FEBS Lett. 2004 Feb 27;560(1-3):192-8.

Han JH, Lee S, Park YS, Park JS, Kim KY, Lim JS, Oh KS, Yang Y. IFITM6 expression is increased in macrophages of tumor-bearing mice. Oncol Rep. 2011 Feb;25(2):531-6.

Hatakeyama J, Philp D, Hatakeyama Y, Haruyama N, Shum L, Aragon MA, Yuan Z, Gibson CW, Sreenath T, Kleinman HK, Kulkarni AB. Amelogenin-mediated regulation of osteoclastogenesis, and periodontal cell proliferation and migration. J Dent Res. 2006 Feb;85(2):144-9. PubMed PMID: 16434732.

He S, Kumar SR, Zhou P, Krasnoperov V, Ryan SJ, Gill PS, Hinton DR. Soluble EphB4 inhibition of PDGF-induced RPE migration in vitro. Invest Ophthalmol Vis Sci. 2010 Jan;51(1):543-52. Epub 2009 Aug 20. PubMed PMID: 19696168; PubMed Central PMCID: PMC2828363.

Héroult M, Schaffner F, Pfaff D, Prahst C, Kirmse R, Kutschera S, Riedel M, Ludwig T, Vajkoczy P, Graeser R, Augustin HG. EphB4 promotes site-specific metastatic tumor cell dissemination by interacting with endothelial cell-expressed ephrinb2. Mol Cancer Res. 2010 Oct;8(10):1297-309.

Hoeger H, Labudova O, Mosgoeller W, Herrera-Marschitz M, Fuerst G, Kitzmüller E, Lubec B. Deficient transcription of subunit RPA 40 of RNA polymerase I and III in heart of rats with neonatal asphyxia. Life Sci. 1998;62(4):275-82. PubMed PMID: 9450498.

Horio T, Kohno M, Kano H, Ikeda M, Yasunari K, Yokokawa ;K,; Minami M, Takeda T. Adrenomedullin as a novel antimigration factor of vascular smooth muscle cells. Circ Res. 1995 Oct;77(4):660-4. PubMed PMID: 7554110.

Huang W, Wang L, Yuan M, Ma J, Hui Y. Adrenomedullin affects two signal transduction pathways and the migration in retinal pigment epithelial cells. Invest Ophthalmol Vis Sci. 2004 May;45(5):1507-13. PubMed PMID: 15111609.

Hugo HJ, Wafai R, Blick T, Thompson EW, Newgreen DF. Staurosporine augments EGF-mediated EMT in PMC42-LA cells through actin depolymerisation, focal contact size reduction and Snail1 induction - a model for cross-modulation. BMC Cancer. 2009 Jul 15;9:235. PubMed PMID: 19604397; PubMed Central PMCID: PMC2717979.

Inaba M, Yuan H, Salzmann V, Fuller MT, Yamashita YM. E-cadherin is required for centrosome and spindle orientation in Drosophila male germline stem cells. PLoS One. 2010 Aug 31;5(8):e12473. PubMed PMID: 20824213; PubMed Central PMCID: PMC2930853.

Inatome R, Yanagi S, Takano T, Yamamura H. A critical role for Syk in endothelial cell proliferation and migration. Biochem Biophys Res Commun. 2001 Aug 10;286(1):195-9. PubMed PMID: 11485328.

Ishiguro T, Avila H, Lin SY, Nakamura T, Yamamoto M, Boyd DD. Gene trapping identifies chloride channel 4 as a novel inducer of colon cancer cell migration, invasion and metastases. Br J Cancer. 2010 Feb 16;102(4):774-82. Epub 2010 Jan 19. PubMed PMID: 20087350; PubMed Central PMCID: PMC2837579.

Ito Y, Oike Y, Yasunaga K, Hamada K, Miyata K, Matsumoto S, Sugano S, Tanihara H, Masuho Y, Suda T. Inhibition of angiogenesis and vascular leakiness by angiopoietin-related protein 4. Cancer Res. 2003 Oct 15;63(20):6651-7. PubMed PMID: 14583458.

Jackson TR, Brown FD, Nie Z, Miura K, Foroni L, Sun J, Hsu VW, Donaldson JG, Randazzo PA. ACAPs are arf6 GTPase-activating proteins that function in the cell periphery. J Cell Biol. 2000 Oct 30;151(3):627-38. PubMed PMID: 11062263; PubMed Central PMCID: PMC2185579.

Jin D, Otani K, Yamahara K, Ikeda T, Nagaya N, Kangawa K. Adrenomedullin reduces expression of adhesion molecules on lymphatic endothelial cells. Regul Pept. 2011 Jan 17;166(1-3):21-7.

Jones KJ, Korb E, Kundel MA, Kochanek AR, Kabraji S, McEvoy M, Shin CY, Wells DG. CPEB1 regulates beta-catenin mRNA translation and cell migration in astrocytes. Glia. 2008 Oct;56(13):1401-13. PubMed PMID: 18618654; PubMed Central PMCID: PMC3013359.

Kanematsu A, Ramachandran A, Adam RM. GATA-6 mediates human bladder smooth muscle differentiation: involvement of a novel enhancer element in regulating alpha-smooth muscle actin gene expression. Am J Physiol Cell Physiol. 2007 Sep;293(3):C1093-102. Epub 2007 Jul 11. PubMed PMID: 17626241.

Karagounis LG, Yaspelkis BB 3rd, Reeder DW, Lancaster GI, Hawley JA, Coffey VG. Contraction-induced changes in TNFalpha and Akt-mediated signalling are associated with increased myofibrillar protein in rat skeletal muscle. Eur J Appl Physiol. 2010 Jul;109(5):839-48. Epub 2010 Mar 13. PubMed PMID: 20229019.

Kato A, Okamoto O, Ishikawa K, Sumiyoshi H, Matsuo N, Yoshioka H, Nomizu M, Shimada T, Fujiwara S. Dermatopontin interacts with fibronectin, promotes fibronectin fibril formation, and enhances cell adhesion. J Biol Chem. 2011 Apr 29;286(17):14861-9.

Kato Y, Kaneko MK, Kunita A, Ito H, Kameyama A, Ogasawara S, Matsuura N, Hasegawa Y, Suzuki-Inoue K, Inoue O, Ozaki Y, Narimatsu H. Molecular analysis of the pathophysiological binding of the platelet aggregation-inducing factor podoplanin to the C-type lectin-like receptor CLEC-2. Cancer Sci. 2008 Jan;99(1):54-61.

Ketterer K, Kong B, Frank D, Giese NA, Bauer A, Hoheisel J, Korc M, Kleeff J, Michalski CW, Friess H. Neuromedin U is overexpressed in pancreatic cancer and increases invasiveness via the hepatocyte growth factor c-Met pathway. Cancer Lett. 2009 May 8;277(1):72-81. Epub 2008 Dec 31. PubMed PMID: 19118941.

Kim SY, Mo JW, Han S, Choi SY, Han SB, Moon BH, Rhyu IJ, Sun W, Kim H. The expression of non-clustered protocadherins in adult rat hippocampal formation and the connecting brain regions. Neuroscience. 2010 Sep 29;170(1):189-99.

Kim SY, Yasuda S, Tanaka H, Yamagata K, Kim H. Non-clustered protocadherin. Cell Adh Migr. 2011 Mar-Apr;5(2):97-105. Epub 2011 Mar 1.

Kim W, Moon SO, Lee S, Sung MJ, Kim SH, Park SK. Adrenomedullin reduces VEGF-induced endothelial adhesion molecules and adhesiveness through a phosphatidylinositol 3'-kinase pathway. Arterioscler Thromb Vasc Biol. 2003 Aug 1;23(8):1377-83.

Kimura M, Kato Y, Sano D, Fujita K, Sakakibara A, Kondo N, Mikami Y, Tsukuda M. Soluble form of ephrinB2 inhibits xenograft growth of squamous cell carcinoma of the head and neck. Int J Oncol. 2009 Feb;34(2):321-7. PubMed PMID: 19148465.

Kohno M, Yasunari K, Minami M, Kano H, Maeda K, Mandal AK, Inoki K, Haneda M, Yoshikawa J. Regulation of rat mesangial cell migration by platelet-derived growth factor, angiotensin II, and adrenomedullin. J Am Soc Nephrol. 1999 Dec;10(12):2495-502. PubMed PMID: 10589687.

Kohno M, Yokokawa K, Kano H, Yasunari K, Minami M, Hanehira T, Yoshikawa J. Adrenomedullin is a potent inhibitor of angiotensin II-induced migration of human coronary artery smooth muscle cells. Hypertension. 1997 Jun;29(6):1309-13. PubMed PMID: 9180634.

Kojima H, Sakurai S, Uemura M, Kitamura K, Kanno H, Nakai Y, Fukui H. Disturbed colocalization of multidrug resistance protein 2 and radixin in human cholestatic liver diseases. J Gastroenterol Hepatol. 2008 Jul;23(7 Pt 2):e120-8. Epub 2007 Aug 28. PubMed PMID: 17725603.

Kotaka M, Kostin S, Ngai S, Chan K, Lau Y, Lee SM, Li H, Ng EK, Schaper J, Tsui SK, Fung K, Lee C, Waye MM. Interaction of hCLIM1, an enigma family protein, with alpha-actinin 2. J Cell Biochem. 2000 Jun 12;78(4):558-65. PubMed PMID:10861853.

Kotaka M, Lau YM, Cheung KK, Lee SM, Li HY, Chan WY, Fung KP, Lee CY, Waye MM, Tsui SK. Elfin is expressed during early heart development. J Cell Biochem. 2001 Aug 21-Sep 5;83(3):463-72. PubMed PMID: 11596114.

Kubo M, Umemoto S, Fujii K, Itoh S, Tanaka M, Kawahara S, Matsuzaki M. Effects of angiotensin II type 1 receptor antagonist on smooth muscle cell phenotype in intramyocardial arteries from spontaneously hypertensive rats. Hypertens Res. 2004 Sep;27(9):685-93. PubMed PMID: 15750263.

Kusumi N, Watanabe M, Yamada H, Li SA, Kashiwakura Y, Matsukawa T, Nagai A, Nasu Y, Kumon H, Takei K. Implication of amphiphysin 1 and dynamin 2 in tubulobulbar complex formation and spermatid release. Cell Struct Funct. 2007;32(2):101-13. Epub 2007 Aug 31. PubMed PMID: 17785912.

Lai KO, Ip FC, Cheung J, Fu AK, Ip NY. Expression of Eph receptors in skeletal muscle and their localization at the neuromuscular junction. Mol Cell Neurosci. 2001 Jun;17(6):1034-47. PubMed PMID: 11414792.

Lake AC, Bialik A, Walsh K, Castellot JJ Jr. CCN5 is a growth arrest-specific gene that regulates smooth muscle cell proliferation and motility. Am J Pathol. 2003 Jan;162(1):219-31. PubMed PMID: 12507905; PubMed Central PMCID: PMC1851113.

Landells LJ, Szilagy CM, Jones NA, Banner KH, Allen JM, Doherty A, O'Connor BJ, pina D, Page CP. Identification and quantification of phosphodiesterase 4 subtypes in CD4 and CD8 lymphocytes from healthy and asthmatic subjects. 2. Br J Pharmacol. 2001 Jul;133(5):722-9.

Lee CK, Lee HM, Kim HJ, Park HJ, Won KJ, Roh HY, Choi WS, Jeon BH, Park TK, Kim B. Syk contributes to PDGF-BB-mediated migration of rat aortic smooth muscle cells via MAPK pathways. Cardiovasc Res. 2007 Apr 1;74(1):159-68. Epub 2007 Jan 18. PubMed PMID: 17303097

Lee HM, Kim HJ, Park HJ, Won KJ, Kim J, Shin HS, Park PJ, Kim HJ, Lee KY, Park SH, Lee CK, Kim B. Spleen tyrosine kinase participates in Src-mediated migration and proliferation by PDGF-BB in rat aortic smooth muscle cells. Arch Pharm Res. 2007 Jun;30(6):761-9. PubMed PMID: 17679556.;

Lee HW, Choi J, Shin H, Kim K, Yang J, Na M, Choi SY, Kang GB, Eom SH, Kim H, Kim E. Preso, a novel PSD-95-interacting FERM and PDZ domain protein thatregulates dendritic spine morphogenesis. J Neurosci. 2008 Dec 31;28(53):14546-56. PubMed PMID: 19118189.

Lee JN, Lian JD, Lee JH, Chard T. Placental proteins (human chorionic gonadotropin, human placental lactogen, pregnancy-specific beta 1-glycoprotein, and placental protein 5) in seminal plasma of normal men and patients with infertility. Fertil Steril. 1983 May;39(5):704-6. PubMed PMID: 6601587.

Leppäranta O, Pulkkinen V, Koli K, Vähätalo R, Salmenkivi K, Kinnula VL, Heikinheimo M, Myllärniemi M. Transcription factor GATA-6 is expressed inquiescent myofibroblasts in idiopathic pulmonary fibrosis. Am J Respir Cell Mol Biol. 2010 May;42(5):626-32. Epub 2009 Jul 13. PubMed PMID: 19597127.

Leterrier JF, Janmey PA, Eyer J. Microtubule-independent regulation of neurofilament interactions in vitro by neurofilament-bound ATPase activities. Biochem Biophys Res Commun. 2009 Jun 19;384(1):37-42. Epub 2009 Apr 18. PubMed PMID: 19379708.

Li X, Tedder TF. CHST1 and CHST2 sulfotransferases expressed by human vascular endothelial cells: cDNA cloning, expression, and chromosomal localization. Genomics. 1999 Feb 1;55(3):345-7.

Li X, Tu L, Murphy PG, Kadono T, Steeber DA, Tedder TF. CHST1 and CHST2 sulfotransferase expression by vascular endothelial cells regulates shear-resistant leukocyte rolling via L-selectin. J Leukoc Biol. 2001 Apr;69(4):565-74.

Luangdilok S, Box C, Patterson L, Court W, Harrington K, Pitkin L, Rhŷs-Evans P, O-charoenrat P, Eccles S. Syk tyrosine kinase is linked to cell motility and progression in squamous cell carcinomas of the head and neck. Cancer Res. 2007 Aug 15;67(16):7907-16. PubMed PMID: 1769979

Ma Z, Nie Z, Luo R, Casanova JE, Ravichandran KS. Regulation of Arf6 and ACAP1 signaling by the PTB-domain-containing adaptor protein GULP. Curr Biol. 2007 Apr 17;17(8):722-7.

McClatchey AI, Fehon RG. Merlin and the ERM proteins--regulators of receptor distribution and signaling at the cell cortex. Trends Cell Biol. 2009May;19(5):198-206. Epub 2009 Apr 1. Review. PubMed PMID: 19345106; PubMed Central PMCID: PMC2796113.

Messina S, Onofri F, Bongiorno-Borbone L, Giovedì S, Valtorta F, Girault JA, Benfenati F. Specific interactions of neuronal focal adhesion kinase isoforms with Src kinases and amphiphysin. J Neurochem. 2003 Jan;84(2):253-65.

Mitch WE, Hu Z, Lee SW, Du J. Strategies for suppressing muscle atrophy in chronic kidney disease: mechanisms activating distinct proteolytic systems. J Ren Nutr. 2005 Jan;15(1):23-7. PubMed PMID: 15648002.

Miyashita K, Itoh H, Sawada N, Fukunaga Y, Sone M, Yamahara K, Yurugi T, Nakao K. Adrenomedullin promotes proliferation and migration of cultured endothelial cells. Hypertens Res. 2003 Feb;26 Suppl:S93-8. PubMed PMID: 12630817.

Mócsai A, Ruland J, Tybulewicz VL. The SYK tyrosine kinase: a crucial player in diverse biological functions. Nat Rev Immunol. 2010 Jun;10(6):387-402. Review.

Montross WT, Ji H, McCrea PD. A beta-catenin/engrailed chimera selectively suppresses Wnt signaling. J Cell Sci. 2000 May;113 ( Pt 10):1759-70. Erratum in: J Cell Sci. 2005 Nov 15;118(Pt 22):5405.

Moreb JS, Baker HV, Chang LJ, Amaya M, Lopez MC, Ostmark B, Chou W. ALDH isozymes downregulation affects cell growth, cell motility and gene expression in lung cancer cells. Mol Cancer. 2008 Nov 24;7:87. PubMed PMID: 19025616; PubMed Central PMCID: PMC2605459.

Nagase T, Kikuno R, Hattori A, Kondo Y, Okumura K, Ohara O. Prediction of the coding sequences of unidentified human genes. XIX. The complete sequences of 100 new cDNA clones from brain which code for large proteins in vitro. DNA Res. 2000 Dec 31;7(6):347-55. PubMed PMID: 11214970.

Nakada M, Niska JA, Miyamori H, McDonough WS, Wu J, Sato H, Berens ME. The phosphorylation of EphB2 receptor regulates migration and invasion of human glioma cells. Cancer Res. 2004 May 1;64(9):3179-85. PubMed PMID: 15126357.

Nakashima K, Yakabe Y. AMPK activation stimulates myofibrillar protein degradation and expression of atrophy-related ubiquitin ligases by increasing FOXO transcription factors in C2C12 myotubes. Biosci Biotechnol Biochem. 2007 Jul;71(7):1650-6. Epub 2007 Jul 7. Erratum in: Biosci Biotechnol Biochem. 2008 Jun;72(6):2008E2. PubMed PMID: 17617726.

Nakatsukasa M, Kawasaki S, Yamasaki K, Fukuoka H, Matsuda A, Tsujikawa M, Tanioka H, Nagata-Takaoka M, Hamuro J, Kinoshita S. Tumor-associated calcium signal transducer 2 is required for the proper subcellular localization of claudin 1 and 7: implications in the pathogenesis of gelatinous drop-like corneal dystrophy. Am J Pathol. 2010 Sep;177(3):1344-55.

Neuhaus B, Bühren S, Böck B, Alves F, Vogel WF, Kiefer F. Migration inhibition of mammary epithelial cells by Syk is blocked in the presence of DDR1 receptors. Cell Mol Life Sci. 2011 Nov;68(22):3757-70. Epub 2011 Apr 17.

Niehrs C, Keller R, Cho KW, De Robertis EM. The homeobox gene goosecoid controls cell migration in Xenopus embryos. Cell. 1993 Feb 26;72(4):491-503. PubMed PMID:8095000.

Noren NK, Yang NY, Silldorff M, Mutyala R, Pasquale EB. Ephrin-independent regulation of cell substrate adhesion by the EphB4 receptor. Biochem J. 2009 Aug 27;422(3):433-42.

Okamoto O, Fujiwara S. Dermatopontin, a novel player in the biology of the extracellular matrix. Connect Tissue Res. 2006;47(4):177-89. Review.

Okamoto O, Hozumi K, Katagiri F, Takahashi N, Sumiyoshi H, Matsuo N, Yoshioka H, Nomizu M, Fujiwara S. Dermatopontin promotes epidermal keratinocyte adhesion via alpha3beta1 integrin and a proteoglycan receptor. Biochemistry. 2010 Jan 12;49(1):147-55.

Otey CA, Dixon R, Stack C, Goicoechea SM. Cytoplasmic Ig-domain proteins: cytoskeletal regulators with a role in human disease. Cell Motil Cytoskeleton. 2009 Aug;66(8):618-34. Review. PubMed PMID: 19466753; PubMed Central PMCID: PMC2735333.

Otey CA, Rachlin A, Moza M, Arneman D, Carpen O. The palladin/myotilin/myopalladin family of actin-associated scaffolds. Int Rev Cytol. 2005;246:31-58. Review. PubMed PMID: 16164966.

Otsuka A, Hirose K, Kilimann MW, Kamata T. Amphiphysin1 inhibits vitronectin-mediated cell adhesion, spreading, and migration in vitro. BiochemBiophys Res Commun. 2003 Feb 14;301(3):769-75. PubMed PMID: 12565847.

Ozaki Y, Suzuki-Inoue K, Inoue O. Novel interactions in platelet biology: CLEC-2/podoplanin and laminin/GPVI. J Thromb Haemost. 2009 Jul;7 Suppl 1:191-4.Review.

Pala D, Kapoor M, Woods A, Kennedy L, Liu S, Chen S, Bursell L, Lyons KM, Carter DE, Beier F, Leask A. Focal adhesion kinase/Src suppresses early chondrogenesis: central role of CCN2. J Biol Chem. 2008 Apr 4;283(14):9239-47.

Pavlik LL, Bezgina EN, Shubina VS, Shatalin YV, Potselueva MM, Moshkov DA. Changes in the ultrastructure and function of goldfish Mauthner neurons in the presence of 3,4-dihydro-2(1H)-pyrimidinethione. Neurosci Behav Physiol. 2008 Feb;38(2):151-5. PubMed PMID: 18197381.

Pearce G, Audzevich T, Jessberger R. SYK regulates B-cell migration by phosphorylation of the F-actin interacting protein SWAP-70. Blood. 2011 Feb3;117(5):1574-84. Epub 2010 Dec 1. PubMed PMID: 21123826.

Pfaff D, Héroult M, Riedel M, Reiss Y, Kirmse R, Ludwig T, Korff T, Hecker M, Augustin HG. Involvement of endothelial ephrin-B2 in adhesion and transmigration of EphB-receptor-expressing monocytes. J Cell Sci. 2008 Nov 15;121(Pt22):3842-50.

Phillips TA, Banerjee SK. CCN5/WISP-2 expression in breast adenocarcinoma is associated with less frequent progression of the disease and suppresses the invasive phenotypes of tumor cells. Cancer Res. 2008 Sep 15;68(18):7606-12. PubMed PMID: 18794149.

Pollitt AY, Grygielska B, Leblond B, Désiré L, Eble JA, Watson SP. Phosphorylation of CLEC-2 is dependent on lipid rafts, actin polymerization, secondary mediators, and Rac. Blood. 2010 Apr 8;115(14):2938-46.

Regulation of smooth muscle cell differentiation by AT-rich interaction domain transcription factors Mrf2alpha and Mrf2beta. Watanabe M, Layne MD, Hsieh CM, Maemura K, Gray S, Lee ME, Jain MK.

Rybalkin SD, Yan C, Bornfeldt KE, Beavo JA. Cyclic GMP phosphodiesterases and regulation of smooth muscle function. Circ Res. 2003 Aug 22;93(4):280-91. Review.

Sacheck JM, Ohtsuka A, McLary SC, Goldberg AL. IGF-I stimulates muscle growth by suppressing protein breakdown and expression of atrophy-related ubiquitin ligases, atrogin-1 and MuRF1. Am J Physiol Endocrinol Metab. 2004 Oct;287(4):E591-601. Epub 2004 Apr 20. PubMed PMID: 15100091.

Sahin M, Greer PL, Lin MZ, Poucher H, Eberhart J, Schmidt S, Wright TM, Shamah SM, O'connell S, Cowan CW, Hu L, Goldberg JL, Debant A, Corfas G, Krull CE, Greenberg ME. Eph-dependent tyrosine phosphorylation of ephexin1 modulates growth cone collapse. Neuron. 2005 Apr 21;46(2):191-204. PubMed PMID: 15848799.

Sakamoto H, Zhang XQ, Suenobu S, Ohbo K, Ogawa M, Suda T. Cell adhesion to ephrinb2 is induced by EphB4 independently of its kinase activity. Biochem Biophys Res Commun. 2004 Aug 27;321(3):681-7.

Sakamoto N, Ishibashi T, Sugimoto K, Sawamura T, Sakamoto T, Inoue N, Saitoh S, Kamioka M, Uekita H, Ohkawara H, Suzuki K, Teramoto T, Maruyama Y, Takeishi Y. Role of LOX-1 in monocyte adhesion-triggered redox, Akt/eNOS and Ca2+ signaling pathways in endothelial cells. J Cell Physiol. 2009 Sep;220(3):706-15.

Schinner E, Salb K, Schlossmann J. Signaling via IRAG is essential for NO/cGMP-dependent inhibition of platelet activation. Platelets. 2011;22(3):217-27.

Schmalbach CE, Chepeha DB, Giordano TJ, Rubin MA, Teknos TN, Bradford CR, Wolf GT, Kuick R, Misek DE, Trask DK, Hanash S. Molecular profiling and the identification of genes associated with metastatic oral cavity/pharynx squamous cell carcinoma. Arch Otolaryngol Head Neck Surg. 2004 Mar;130(3):295-302.

Schulz WA, Alexa A, Jung V, Hader C, Hoffmann MJ, Yamanaka M, Fritzsche S, Wlazlinski A, Müller M, Lengauer T, Engers R, Florl AR, Wullich B, Rahnenführer J. Factor interaction analysis for chromosome 8 and DNA methylation alterations highlights innate immune response suppression and cytoskeletal changes in prostate cancer. Mol Cancer. 2007 Feb 5;6:14. PubMed PMID: 17280610; PubMed Central PMCID: PMC1797054.

Schulz WA, Ingenwerth M, Djuidje CE, Hader C, Rahnenführer J, Engers R. Changes in cortical cytoskeletal and extracellular matrix gene expression in prostate cancer are related to oncogenic ERG deregulation. BMC Cancer. 2010 Sep22;10:505. PubMed PMID: 20860828; PubMed Central PMCID: PMC2955608.

Schymeinsky J, Then C, Sindrilaru A, Gerstl R, Jakus Z, Tybulewicz VL, Scharffetter-Kochanek K, Walzog B. Syk-mediated translocation of PI3Kdelta to the leading edge controls lamellipodium formation and migration of leukocytes. PLoS One. 2007 Nov 7;2(11):e1132. PubMed PMID: 17987119; PubMed Central PMCID: PMC2063580.;

Schymeinsky J, Then C, Walzog B. The non-receptor tyrosine kinase Syk regulates lamellipodium formation and site-directed migration of humanleukocytes. J Cell Physiol. 2005 Aug;204(2):614-22. PubMed PMID: 15754322.

Sehic A, Risnes S, Khan QE, Khuu C, Osmundsen H. Gene expression and dental enamel structure in developing mouse incisor. Eur J Oral Sci. 2010Apr;118(2):118-30. PubMed PMID: 20487000.

Senior PV, Zhang BX, Chan ST. Loss of cell-surface receptor EphB2 is important for the growth, migration, and invasiveness of a colon cancer cell line. Int J Colorectal Dis. 2010 Jun;25(6):687-94. Epub 2010 Mar 26. PubMed PMID: 20339854.;

Shamah SM, Lin MZ, Goldberg JL, Estrach S, Sahin M, Hu L, Bazalakova M, Neve RL, Corfas G, Debant A, Greenberg ME. EphA receptors regulate growth cone dynamics through the novel guanine nucleotide exchange factor ephexin. Cell. 2001 Apr 20;105(2):233-44. PubMed PMID: 11336673.

Shan L, Yu M, Snyderwine EG. Global gene expression profiling of chemically induced rat mammary gland carcinomas and adenomas. Toxicol Pathol. 2005;33(7):768-75.

Smeds E, Habuchi H, Do AT, Hjertson E, Grundberg H, Kimata K, Lindahl U, Kusche-Gullberg M. Substrate specificities of mouse heparan sulphate glucosaminyl 6-O-sulphotransferases. Biochem J. 2003 Jun 1;372(Pt 2):371-80. PubMed PMID: 12611590; PubMed Central PMCID: PMC1223407.

Snow AD, Nochlin D, Sekiguichi R, Carlson SS. Identification in immunolocalization of a new class of proteoglycan (keratan sulfate) to the neuritic plaques of Alzheimer's disease. Exp Neurol. 1996 Apr;138(2):305-17. PubMed PMID: 8620929.

Sokol SY, Melton DA. Interaction of Wnt and activin in dorsal mesoderm induction in Xenopus. Dev Biol. 1992 Dec;154(2):348-55. PubMed PMID: 1426642.

Speirs CK, Jernigan KK, Kim SH, Cha YI, Lin F, Sepich DS, DuBois RN, Lee E, Solnica-Krezel L. Prostaglandin Gbetagamma signaling stimulates gastrulation movements by limiting cell adhesion through Snai1a stabilization. Development. 2010 Apr;137(8):1327-37.

Stein E, Lane AA, Cerretti DP, Schoecklmann HO, Schroff AD, Van Etten RL, Daniel TO. Eph receptors discriminate specific ligand oligomers to determine alternative signaling complexes, attachment, and assembly responses. Genes Dev. 1998 Mar 1;12(5):667-78. PubMed PMID: 17454677.

Stevens NR, Raposo AA, Basto R, St Johnston D, Raff JW. From stem cell to embryo without centrioles. Curr Biol. 2007 Sep 4;17(17):1498-503. Epub 2007 Aug 23. PubMed PMID: 17716897; PubMed Central PMCID: PMC1971134.

Stimamiglio MA, Jiménez E, Silva-Barbosa SD, Alfaro D, García-Ceca JJ, Muñoz JJ, Cejalvo T, Savino W, Zapata A. EphB2-mediated interactions are essential for proper migration of T cell progenitors during fetal thymus colonization. J Leukoc Biol. 2010 Sep;88(3):483-94. Epub 2010 May 26. PubMed PMID: 20504947.;

Storbeck CJ, Wagner S, O'Reilly P, McKay M, Parks RJ, Westphal H, Sabourin LA.; Hägg S, Skogsberg J, Lundström J, Noori P, Nilsson R, Zhong H, Maleki S, Shang MM, Brinne B, Bradshaw M, Bajic VB, Samnegård A, Silveira A, Kaplan LM, Gigante B, Leander K, de Faire U, Rosfors S, Lockowandt U, Liska J, Konrad P, Takolander R, Franco-Cereceda A, Schadt EE, Ivert T, Hamsten A, Tegnér J, Björkegren J. Multi-organ xpression profiling uncovers a gene module in coronary artery disease involving transendothelial migration of leukocytes and LIM domain binding2: the Stockholm Atherosclerosis Gene Expression (STAGE) study. PLoS Genet. 2009 Dec;5(12):e1000754. Epub 2009 Dec 4. PubMed PMID: 19997623; PubMed Central PMCID:PMC2780352.

Tabata T, Sakaguchi K, Tajima T, Suzuki AS. Comparative study of sequential expression of the organizer-related genes in normal Cynops pyrrhogaster embryos and mesodermalized ectoderm. Dev Growth Differ. 2001 Aug;43(4):351-9. PubMed PMID: 11473542.

Takeda U, Utani A, Wu J, Adachi E, Koseki H, Taniguchi M, Matsumoto T, Ohashi T, Sato M, Shinkai H. Targeted disruption of dermatopontin causes abnormal collagen fibrillogenesis. J Invest Dermatol. 2002 Sep;119(3):678-83.

Tanaka SS, Yamaguchi YL, Tsoi B, Lickert H, Tam PP. IFITM/Mil/fragilis family proteins IFITM1 and IFITM3 play distinct roles in mouse primordial germ cell homing and repulsion. Dev Cell. 2005 Dec;9(6):745-56.

Tatenhorst L, Senner V, Püttmann S, Paulus W. Regulators of G-protein signaling 3 and 4 (RGS3, RGS4) are associated with glioma cell motility. J Neuropathol Exp Neurol. 2004 Mar;63(3):210-22. PubMed PMID: 15055445.

Taylor AC, Murfee WL, Peirce SM. EphB4 expression along adult rat microvascular networks: EphB4 is more than a venous specific marker.Microcirculation. 2007 Apr-May;14(3):253-67. PubMed PMID: 17454677.

Tirumala Vani G, Mukesh N, Siva Prasad B, Rama Devi P, Hema Prasad M, Usha Rani P, Pardhanandana Reddy P. Role of glutathione S-transferase Mu-1 (GSTM1) polymorphism in oligospermic infertile males. Andrologia. 2010 Aug;42(4):213-7. PubMed PMID: 20629642.

Truitt L, Freywald T, DeCoteau J, Sharfe N, Freywald A. The EphB6 receptor cooperates with c-Cbl to regulate the behavior of breast cancer cells. Cancer Res. 2010 Feb 1;70(3):1141-53. Epub 2010 Jan 19. PubMed PMID: 20086179.

Tsukita S, Furuse M. The structure and function of claudins, cell adhesion molecules at tight junctions. Ann N Y Acad Sci. 2000;915:129-35. Review.

Umar A, Luider TM, Berrevoets CA, Grootegoed JA, Brinkmann AO. Proteomic analysis of androgen-regulated protein expression in a mouse fetal vas deferens cell line. Endocrinology. 2003 Apr;144(4):1147-54. PubMed PMID: 12639895.

Umetani M, Mataki C, Minegishi N, Yamamoto M, Hamakubo T, Kodama T. Function of GATA transcription factors in induction of endothelial vascular cell adhesion molecule-1 by tumor necrosis factor-alpha. Arterioscler Thromb Vasc Biol. 2001 Jun;21(6):917-22.

Usui T, Kazami S, Dohmae N, Mashimo Y, Kondo H, Tsuda M, Terasaki AG, Ohashi K, Kobayashi J, Osada H. Amphidinolide h, a potent cytotoxic macrolide, covalently binds on actin subdomain 4 and stabilizes actin filament. Chem Biol. 2004 Sep;11(9):1269-77. PubMed PMID: 15380187.

Vergaño-Vera E, Fernández AP, Hurtado-Chong A, Vicario-Abejón C, Martínez A. Lack of adrenomedullin affects growth and differentiation of adult neural stem/progenitor cells. Cell Tissue Res. 2010 Apr;340(1):1-11. Epub 2010 Feb 25. PubMed PMID: 20182890.

Villablanca EJ, Renucci A, Sapède D, Lec V, Soubiran F, Sandoval PC, Dambly-Chaudière C, Ghysen A, Allende ML. Control of cell migration in thezebrafish lateral line: implication of the gene "tumour-associated calcium signal transducer," tacstd. Dev Dyn. 2006 Jun;235(6):1578-88. PubMed PMID: 16552761.

Villone G, Veneziani BM, Picone R, De Amicis F, Perrotti N, Tramontano D. In the thyroid cells proliferation, differentiated and metabolic functions are under the control of different steps of the cyclic AMP cascade. Mol Cell Endocrinol. 1993 Sep;95(1-2):85-93. PubMed PMID: 8243811.

Wacker S, Brodbeck A, Lemaire P, Niehrs C, Winklbauer R. Patterns and control of cell motility in the Xenopus gastrula. Development. 1998 May;125(10):1931-42.

Wada H, Abe M, Ono K, Morimoto T, Kawamura T, Takaya T, Satoh N, Fujita M, Kita T, Shimatsu A, Hasegawa K. Statins activate GATA-6 and induce differentiated vascular smooth muscle cells. Biochem Biophys Res Commun. 2008 Oct 3;374(4):731-6. Epub 2008 Jul 29. PubMed PMID: 18671946.

Wada H, Hasegawa K, Morimoto T, Kakita T, Yanazume T, Abe M, Sasayama S. Calcineurin-GATA-6 pathway is involved in smooth muscle-specific transcription. J Cell Biol. 2002 Mar 18;156(6):983-91. Epub 2002 Mar 11. PubMed PMID: 11889139; PubMed Central PMCID: PMC2173472.

Watson AA, Brown J, Harlos K, Eble JA, Walter TS, O'Callaghan CA. The crystal structure and mutational binding analysis of the extracellular domain of the platelet-activating receptor CLEC-2. J Biol Chem. 2007 Feb 2;282(5):3165-72.

Wilson KA, Colavito S, Schulz V, Wakefield PH, Sessa W, Tuck DP, Stern DF. NFBD1/MDC1 Regulates Cav1 and Cav2 independently of DNA Damage and p53. Mol Cancer Res. 2011 May 6. [Epub ahead of print]

Winning RS, Ward EK, Scales JB, Walker GK. EphA4 catalytic activity causes inhibition of RhoA GTPase in Xenopus laevis embryos. Differentiation. 2002 Mar;70(1):46-55. PubMed PMID: 11963655.

Xia CF, Yin H, Borlongan CV, Chao J, Chao L. Adrenomedullin gene delivery protects against cerebral ischemic injury by promoting astrocyte migration and survival. Hum Gene Ther. 2004 Dec;15(12):1243-54. PubMed PMID: 15684700.

Xu X, Dong C, Vogel BE. Hemicentins assemble on diverse epithelia in the mouse. J Histochem Cytochem. 2007 Feb;55(2):119-26.

Xu X, Mannik J, Kudryavtseva E, Lin KK, Flanagan LA, Spencer J, Soto A, Wang N, Lu Z, Yu Z, Monuki ES, Andersen B. Co-factors of LIM domains (Clims/Ldb/Nli) regulate corneal homeostasis and maintenance of hair follicle stem cells. DevBiol. 2007 Dec 15;312(2):484-500.

Xu X, Vogel BE. A secreted protein promotes cleavage furrow maturation during cytokinesis. Curr Biol. 2011 Jan 25;21(2):114-9.

Yamada H, Ohashi E, Abe T, Kusumi N, Li SA, Yoshida Y, Watanabe M, Tomizawa K, Kashiwakura Y, Kumon H, Matsui H, Takei K. Amphiphysin 1 is important for actin polymerization during phagocytosis. Mol Biol Cell. 2007 Nov;18(11):4669-80. Epub 2007 Sep 12. PubMed PMID: 17855509; PubMed Central PMCID: PMC2043535.

Yamanaka Y, Mammoto T, Kirita T, Mukai M, Mashimo T, Sugimura M, Kishi Y, Nakamura H. Epinephrine inhibits invasion of oral squamous carcinoma cells by modulating intracellular cAMP. Cancer Lett. 2002 Feb 25;176(2):143-8. PubMed PMID: 11804741.

Yaroslavskiy BB, Turkova I, Wang Y, Robinson LJ, Blair HC. Functional osteoclast attachment requires inositol-1,4,5-trisphosphate receptor-associated cGMP-dependent kinase substrate. Lab Invest. 2010 Oct;90(10):1533-42.

Yin F, Herring BP. GATA-6 can act as a positive or negative regulator of smooth muscle-specific gene expression. J Biol Chem. 2005 Feb 11;280(6):4745-52. Epub 2004 Nov 18. PubMed PMID: 15550397.

Yu F, Ng SS, Chow BK, Sze J, Lu G, Poon WS, Kung HF, Lin MC. Knockdown of interferon-induced transmembrane protein 1 (IFITM1) inhibits proliferation, migration, and invasion of glioma cells. J Neurooncol. 2011 Jun;103(2):187-95. Epub 2010 Sep 14. PubMed PMID: 20838853.

Yu H, Ye L, Mansel RE, Zhang Y, Jiang WG. Clinical implications of the influence of Ehm2 on the aggressiveness of breast cancer cells through regulation of matrix metalloproteinase-9 expression. Mol Cancer Res. 2010 Nov;8(11):1501-12.

Yu H, Zhang Y, Ye L, Jiang WG. The FERM family proteins in cancer invasion and metastasis. Front Biosci. 2011 Jan 1;16:1536-50. Review.

Zaidel-Bar R, Joyce MJ, Lynch AM, Witte K, Audhya A, Hardin J. The F-BAR domain of SRGP-1 facilitates cell-cell adhesion during C. elegans morphogenesis. J Cell Biol. 2010 Nov 15;191(4):761-9.

Zhang B, Gu F, She C, Guo H, Li W, Niu R, Fu L, Zhang N, Ma Y. Reduction of Akt2 inhibits migration and invasion of glioma cells. Int J Cancer. 2009 Aug 1;125(3):585-95.

Zhang JS, Gong A, Young CY. ZNF185, an actin-cytoskeleton-associated growth inhibitory LIM protein in prostate cancer. Oncogene. 2007 Jan 4;26(1):111-22. Epub 2006 Jun 26.

Zhang T, Zhuang S, Casteel DE, Looney DJ, Boss GR, Pilz RB. A cysteine-rich LIM-only protein mediates regulation of smooth muscle-specific gene expression by cGMP-dependent protein kinase. J Biol Chem. 2007 Nov 16;282(46):33367-80. Epub 2007 Sep 18. PubMed PMID: 17878170.

Zhang X, Shrikhande U, Alicie BM, Zhou Q, Geahlen RL. Role of the protein tyrosine kinase Syk in regulating cell-cell adhesion and motility in breast cancer cells. Mol Cancer Res. 2009 May;7(5):634-44. Epub 2009 May 12. PubMed PMID: 19435818; PubMed Central PMCID: PMC2788757.

Zhao Y, Zhou T, Li A, Yao H, He F, Wang L, Si J. A potential role of collagens expression in distinguishing between premalignant and malignant lesions in stomach. Anat Rec (Hoboken). 2009 May;292(5):692-700.

Zhou HY, Chen WD, Zhu DL, Wu LY, Zhang J, Han WQ, Li JD, Yan C, Gao PJ. The PDE1A-PKCalpha signaling pathway is involved in the upregulation of alpha-smooth muscle actin by TGF-beta1 in adventitial fibroblasts. J Vasc Res. 2010;47(1):9-15.

Zimmermann H. Accumulation of synaptic vesicle proteins and cytoskeletal specializations at the peripheral node of Ranvier. Microsc Res Tech. 1996 Aug 1;34(5):462-73. Review. PubMed PMID: 8837022.

Zou X, Bolon B, Pretorius JK, Kurahara C, McCabe J, Christiansen KA, Sun N, Duryea D, Foreman O, Senaldi G, Itano AA, Siu G. Neonatal death in mice lacking cardiotrophin-like cytokine is associated with multifocal neuronal hypoplasia. Vet Pathol. 2009 May;46(3):514-9. Epub 2008 Dec 19. PubMed PMID: 19098279.
