# Supplementary material for: Uncovering the cellular and molecular changes in tendon stem/progenitor cells attributed to tendon aging and degeneration
Source: Aging Cell. 2013 Jul 22;12(6):988–99. doi: 10.1111/acel.12124 (PMC4225469; doi:10.1111/acel.12124)
Supplement: Supplementary file 10 — Table S6 Differentially expressed genes selected from the microarray data for further validation on RNA or protein level. Table S7 Primer pairs and PCR conditions. Supplementary information of study protocols including detailed information on: Calculation of population doubling and colony-forming unit (CFU assay); FACS and immunocytochemistry protocols; Three-lineage differentiation protocols; RNA isolation, cDNA synthesis and PCR; Microarray analysis; Western blotting protocols. [file acel0012-0988-SD10.doc]

**Table S6. Differentially expressed genes selected from the microarray data for further validation on RNA or protein level.**

| **Gene name** | **Primary function** | **Accession number** | **Mean signal A-TSPC** | **Mean signal Y-TSPC** | **Ratio A-/Y-TSPC** | **Mean of ratios A/Y** | **p-value** | **Mean of p-value** |
| --- | --- | --- | --- | --- | --- | --- | --- | --- |
| **Collagen I α_1_** | Cell substrate | 202311_s_at | 34637.36 | 45131.77 | 0.7675 | 0.8872 | 0.30 | 0.43 |
|  |  | 1556499_s_at | 94493.63 | 97874.88 | 0.9655 |  | 0.30 |  |
|  |  | 217430_x_at | 3651.67 | 4100.47 | 0.8905 |  | 0.55 |  |
|  |  | 202310_s_at | 85059.87 | 91923.94 | 0.9253 |  | 0.55 |  |
| **Collagen I α_2_** |  | 229218_at | 13861.73 | 22325.7 | 0.6209 | 0.8413 | 0.55 | 0.55 |
|  |  | 202404_s_at | 98212.00 | 102949.9 | 0.9540 |  | 0.55 |  |
|  |  | 202403_s_at | 105965.06 | 111669 | 0.9489 |  | 0.55 |  |
| **Fibronectin** |  | 1558199_at | 1403.30 | 1310.07 | 1.0712 | 1.1521 | 0.85 | 0.49 |
|  |  | 210495_x_at | 80081.00 | 75747.76 | 1.0572 |  | 0.30 |  |
|  |  | 212464_s_at | 95553.10 | 93083.04 | 1.0265 |  | 0.85 |  |
|  |  | 211719_x_at | 88924.97 | 87826.63 | 1.0125 |  | 0.55 |  |
|  |  | 214701_s_at | 4767.10 | 3382 | 1.4096 |  | 0.30 |  |
|  |  | 214702_at | 6491.03 | 4600.2 | 1.4110 |  | 0.30 |  |
|  |  | 216442_x_at | 79201.63 | 73552 | 1.0768 |  | 0.30 |  |
| **Integrin α_1_** | Cell adhesion | 214660_at | 623.5 | 712.2 | 0.8755 | 0.9054 | 0.55 | 0.55 |
|  |  | 226731_at | 985.5 | 1053.53 | 0.9354 |  | 0.55 |  |
| **Integrin α_2_** |  | 227314_at | 547.70 | 464.97 | 1.1779 | 1.1779 | 0.55 | 0.55 |
| **Integrin α_5_** |  | 201389_at | 5375.37 | 4101.27 | 1.3107 | 1.3107 | 0.15 | 0.15 |
| **Integrin α_11_** |  | 222899_at | 7500.27 | 10322.27 | 0.7266 | 0.7266 | 0.85 | 0.85 |
|  |  | 1554819_a_at | 506.67 | 717.37 | 0.7063 |  | 0.85 |  |
| **Integrin α_V_** |  | 20235_at | 28228.87 | 25830.33 | 1.0929 | 1.0929 | 0.85 | 0.85 |
| **Integrin β_1_** |  | 211945_s_at | 71370.60 | 75682.27 | 0.9430 | 0.8370 | 0.30 | 0.29 |
|  |  | 216178_x_at | 264.5 | 351 | 0.7536 |  | 0.55 |  |
|  |  | 1553530_a_at | 19589.67 | 24811.27 | 0.7895 |  | 0.15 |  |
|  |  | 1553678_a_at | 33420.80 | 38784.93 | 0.8617 |  | 0.15 |  |
| **Integrin β_3_** |  | 204627_s_at | 282.07 | 288.37 | 0.9782 | 1.3124 | 0.85 | 0.60 |
|  |  | 204628_s_at | 240.07 | 219.3 | 1.0947 |  | 0.85 |  |
|  |  | 215240_at | 96.17 | 67.3 | 1.4290 |  | 0.55 |  |
|  |  | 216261_at | 159.57 | 91.3 | 1.7478 |  | 0.15 |  |
| **Integrin β_5_** |  | 214020_x_at | 2087.93 | 1948.63 | 1.0715 | 1.0493 | 0.15 | 0.53 |
|  |  | 214021_x_at | 1958.7 | 1861.5 | 1.0522 |  | 0.85 |  |
|  |  | 201124_at | 2826.87 | 2648.13 | 1.0675 |  | 0.55 |  |
|  |  | 201125_s_at | 19883.2 | 19768.3 | 1.0058 |  | 0.55 |  |
| **Gene name** | **Primary function** | **Accession number** | **Mean signal A-TSPC** | **Mean signal Y-TSPC** | **Ratio A-/Y-TSPC** | **Mean of ratios A/Y** | **p-value** | **Mean of p-value** |
| **ROCK1** | Protein phosphory-lation | 214578_s_at | 2007.1 | 1670.57 | 1.2014 | 1.1859 | 0.30 | 0.30 |
|  |  | 213044_at | 13802.73 | 11793.37 | 1.1704 |  | 0.30 |  |
| **ROCK2** |  | 202762_at | 7563.67 | 6281.07 | 1.2042 | 1.1284 | 0.55 | 0.55 |
|  |  | 211504_x_at | 492.93 | 468.27 | 1.0527 |  | 0.55 |  |

The table includes data from three Y-TSPC and three A-TSPC donors.

**Table S7**. Primer pairs and PCR conditions.

| **Target gene** | **Primers** | **Annealing temperature [**°**C]** | **Cycle numbers** | **Product size**  **[bp]** | **Reference** |
| --- | --- | --- | --- | --- | --- |
| **Eya1** | F 5´-gttcatctgggacttgga-3´  R 5´-gcttaggtcctgtccgtt-3´ | 46 | 40 | 229 | Okada *et al.* 2006 |
| **Six1** | F 5´-tgcttgttggaggaggagtt-3´  R 5´-aaggagaagtcgaggggtgt-3´ | 55 | 35 | 206 | Ng *et al.* 2006 |
| **Collagen I α_1_** | F 5´-ccagtcacctgcgtacagaa-3´  R 5´-gagaccacgaggaccagaag-3´ | 54 | 40 | 223 | Gronthos *et al.* 2006 |
| **Collagen III** | F 5´-ttataaaccaaactctatct-3´  R 5´-tattatagcaccattgagac-3´ | 42 | 35 | 260 | Boykiw *et al.* 1998 |
| **COMP** | F 5´-gctctgtggcatacaggaga-3´  R 5´-catagaatcgcaccctgatg-3´ | 53 | 40 | 145 | Tian *et al.* 2006 |
| **Decorin** | F 5´-gctggaccgtttcaacagagag-3´  R 5´-tcattctcatgggcacgcag-3´ | 54 | 40 | 437 | Chung *et al.* 2002 |
| **TenascinC** | F 5´-gagaaaggcagacacaagag-3´  R 5´-gcagtccagttgagtttgag-3´ | 57 | 35 | 395 | Self designed |
| **Fibronectin** | F 5´-gctaccttctactgatggcgaatag-3´  R 5´-cctcaattgttgttcgctgga-3´ | 52 | 28 | 83 | Chen *et al.* 2005 |
| **p16^INKA4^** | F 5´-caacgcaccgaatagttacg-3´  R 5´-agcaccaccagcgtgtc-3´ | 57 | 35 | 423 | Frere-Belda *et al.* 2004 |
| **p14^ARF^** | F 5´-gggttttcgtggttcacatc-3´  R 5´-cgctgcccatcatcatgac-3´ | 58 | 35 | 151 |
| **p21^WAF1^** | F 5´-gaacttcgactttgtcaccgag-3´  R 5´-cgttttcgaccctgagagtctc-3´ | 60 | 30 | 279 | Alcantara *et al.* 2001 |
| **p53** | F 5´-aaggaaatttgcgtgtggag-3´  R 5´-ttctgacgcacacctattgc-3´ | 58 | 35 | 702 | Gan *et al.* 2005 |
| **Integrin α5** | F 5´-actaggaaatccattcacagttc-3´  R 5´-gcatagttagtgttctttgttgg-3´ | 52 | 29 | 201 | Lin *et al.* 2005 |
| **Integrin αv** | F 5´-ggagcacatttagttgaggtat -3´  R 5´-actgttgctaggtggtaaaact-3´ | 46 | 28 | 274 |
| **Integrin β1** | F 5´-atgaatgaaatgaggaggattacttcg-3´  R 5´-aaaacaccagcagccgtgtaac-3´ | 52 | 27 | 322 |
| **Integrin β3** | F 5´-ctgctgtagacatttgctatga-3´  R 5´-gccaagaggtagaaggtaaata-3´ | 52 | 35 | 211 |
| **Integrin β5** | F 5´-ctgtggactgatgtttcctt-3´  R 5´-gtatgctggttttacagactcc-3´ | 54 | 29 | 407 |
| **GAPDH** | F 5´-caactacatggtttacatgttc-3´  R 5´-gccagtggactccacgac-3´ | 50 | 30 | 181 | Takahashi *et al.* 2003 |

F – forward primer; R – reverse primer; in PCRs was used diluted 1:10 or 1:5 cDNA.

The primer sets used for quantitative PCR (Scleraxis, tenomodulin, integrins α1, α2 and α11, and HPRT) are not included in the table. Their sequences are property of Search LC GmbH (Heilderberg, Germany) and for further details refer to the primer kit description of the producer.
